# Jasmonates, gibberellins, and powdery mildew modify cell cycle progression and evoke differential spatiotemporal responses along the barley leaf

**DOI:** 10.1093/jxb/erad331

**Published:** 2023-08-23

**Authors:** Jovaras Krasauskas, Showkat Ahmad Ganie, Aroub Al-Husari, Laurence Bindschedler, Pietro Spanu, Masaki Ito, Alessandra Devoto

**Affiliations:** Plant Molecular Science and Centre of Systems and Synthetic Biology, Department of Biological Sciences, Royal Holloway, University of London, Egham, Surrey, TW20 0EX, UK; Plant Molecular Science and Centre of Systems and Synthetic Biology, Department of Biological Sciences, Royal Holloway, University of London, Egham, Surrey, TW20 0EX, UK; Plant Molecular Science and Centre of Systems and Synthetic Biology, Department of Biological Sciences, Royal Holloway, University of London, Egham, Surrey, TW20 0EX, UK; Plant Molecular Science and Centre of Systems and Synthetic Biology, Department of Biological Sciences, Royal Holloway, University of London, Egham, Surrey, TW20 0EX, UK; Department of Life Sciences, Imperial College London, London, SW7 2AZ, UK; School of Biological Science and Technology, Kanazawa University, Ishikawa 920-1192, Japan; Plant Molecular Science and Centre of Systems and Synthetic Biology, Department of Biological Sciences, Royal Holloway, University of London, Egham, Surrey, TW20 0EX, UK; Cardiff University, UK

**Keywords:** Barley, *Blumeria hordei*, cell cycle progression, *Hordeum vulgare*, leaf development, powdery mildew, jasmonates, gibberellins

## Abstract

Barley (*Hordeum vulgare*) is an important cereal crop, and its development, defence, and stress responses are modulated by different hormones including jasmonates (JAs) and the antagonistic gibberellins (GAs). Barley productivity is severely affected by the foliar biotrophic fungal pathogen *Blumeria hordei*. In this study, primary leaves were used to examine the molecular processes regulating responses to methyl-jasmonate (MeJA) and GA to *B. hordei* infection along the leaf axis. Flow cytometry, microscopy, and spatiotemporal expression patterns of genes associated with JA, GA, defence, and the cell cycle provided insights on cell cycle progression and on the gradient of susceptibility to *B. hordei* observed along the leaf. Notably, the combination of *B. hordei* with MeJA or GA pre-treatment had a different effect on the expression patterns of the analysed genes compared to individual treatments. MeJA reduced susceptibility to *B. hordei* in the proximal part of the leaf blade. Overall, distinctive spatiotemporal gene expression patterns correlated with different degrees of cell proliferation, growth capacity, responses to hormones, and *B. hordei* infection along the leaf. Our results highlight the need to further investigate differential spatial and temporal responses to pathogens at the organ, tissue, and cell levels in order to devise effective disease control strategies in crops.

## Teaser

Cellular and gene expression analyses along the barley leaf axis show differential spatiotemporal responses to *Blumeria hordei* associated with the developmental gradient, and are fine-tuned by the phytohormones jasmonates and gibberellins

## Introduction

Barley is a widely grown cereal that is cultivated for animal feed, malting, and human consumption (Akar *et al*., 2004). In plants, growth, development, and stress responses are modulated by different levels and gradients of phytohormones (Kupke *et al.*, 2022; Cao *et al.*, 2017). Numerous studies have shown how such hormones affect the signalling networks that modulate cell division and thus modulate growth and development, including abscisic acid (ABA; Świątek *et al.*, 2002; (Skirycz and Inzé, 2010), auxin (Perrot-Rechenmann, 2010), brassinosteroids (reviewed by Clouse, 2011), cytokinins (reviewed by Haberer and Kieber, 2002), gibberellins (GAs; Achard *et al*., 2009), and jasmonates (JAs, including active precursors and derivatives of jasmonic acid; Noir *et al.*, 2013; Bömer *et al.,* 2018). The levels of stress-related hormones such as JA and salicylic acid (SA) differ along the maize leaf (Nelissen *et al*., 2018), whilst in barley JAs are relatively more abundant in the root tip, the scutella node, and in the leaf base, but not at the leaf tip (Maucher *et al.*, 2000, 2004). These studies therefore also suggest a developmental role for JA and SA.

JAs are oxylipins, which are oxygenated fatty acids synthesized from α-linolenic acid in chloroplast membranes and subsequently oxygenated by lipoxygenases (LOXs) to hydroperoxide derivatives, from which different JA-derived metabolites are formed (reviewed by Wasternack and Song, 2017). The methylated form (MeJA) is the most characterized and stable jasmonate (Wasternack and Hause, 2013; Perez Salamo *et al*., 2019). In addition to their possible role in development as described above, JAs in Arabidopsis have been shown to be key regulators of plant defence or homeostasis in response to pathogens, wounding, and environmental stresses (Balbi and Devoto, 2008; Browse, 2009; Pérez-Salamó *et al.*, 2019). In comparison to maize and rice, less is known about JA biosynthesis and signalling in barley and wheat (Lyons *et al*., 2013). The F-box protein CORONATINE INSENSITIVE1 (COI1; Xie *et al.*, 1998; Devoto *et al.,* 2002; Xu *et al.,* 2002; Pérez-Salamó *et al.*, 2019) acts as a receptor for JAs in Arabidopsis (Thines *et al*., 2007; Chini *et al*., 2007; Katsir *et al*., 2008) and rice (Yan *et al*., 2018). In Arabidopsis, COI1 associates with multiple co-factors to form the SCF^COI1^ complex ,which recruits and ubiquitinates Jasmonate ZIM-domain (JAZ) repressor proteins for degradation to modulate JA responses (Devoto *et al.*, 2002; Xu *et al.*, 2002; reviewed in Pérez-Salamó *et al.*, 2019).

Gibberellins (GAs) derived from gibberellic acid are a large family of tetracyclic diterpenoids, first identified as secondary metabolites in the pathogenic fungus *Gibberella fujikuroi*, and they are ubiquitous in higher plants where they function as endogenous growth regulators (Hedden and Thomas, 2012; Binenbaum *et al*., 2018; Salazar-Cerezo *et al*., 2018). The interaction between GA and JA signalling through DELLA proteins has been extensively reviewed (Navarro *et al*., 2008; Hong *et al*., 2012; Zhong and Yang, 2012; Pérez-Salamó *et al*., 2019). Briefly, GAs play crucial roles in promoting growth and development, notably by triggering the degradation of the growth-repressor DELLA proteins in angiosperms (reviewed by Harberd *et al*., 2009). DELLAs integrate plant hormone signalling pathways and environmental changes through physical interactions with regulatory proteins such as JAZs (Hou *et al.*, 2010; Wild *et al.*, 2012; Yang *et al.*, 2012) and the MYC2 transcription factor (Hong *et al.*, 2012). Similar to the action of JA on JAZs, through its GA-INSENSITIVE DWARF1 (GID1) receptor, GAs recruit DELLA proteins for ubiquitination and degradation via the 26S proteasome, thereby resulting in activation of the GA response (Qi *et al.*, 2014). GA and JA synergistically and antagonistically interact to regulate seedling growth and resistance to pathogens through the interaction between JAZs and DELLAs (Song and Bent, 2014; de Vleesschauwer *et al.*, 2016).

The coordination of cell division and cell expansion are critical to the normal growth and development of plant tissues and organs. The regulation machinery of the cell cycle is tightly temporally and spatially coordinated during plant development by the action of phytohormones (Gonzalez *et al.*, 2010; Dudits *et al.*, 2011; Nelissen *et al.*, 2012, 2018). In monocots, such as maize and wheat, dividing cells are found at the base of the leaf, while expanding and mature cells are located towards the tip (Nelissen *et al*., 2016; Loudya *et al*., 2021). In dicots such as Arabidopsis, cell division continues for longer at the leaf base and ceases earlier at the tip (Nelissen *et al*., 2016). In comparison, the characterization of cell-cycle regulation in barley is less advanced, but several orthologues of cyclins and cyclin-dependent kinases of the G1/S and G2/M transitions are known. As in Arabidopsis, Cyclin Dependent Kinase A1 (CDKA1) contributes to initiating the cell cycle at G1/S as well as being active in the G2/M phase in rice (Umeda *et al.*, 1999; Lee *et al*., 2003; Qu *et al*., 2018), barley (Gendreau *et al*., 2012; Jin *et al*., 2020), and maize (Dante *et al*., 2014). For full CDKA1 activity, the combination with Cyclin Dependent Kinase D1 (CDKD1) is essential (Sofroni *et al.*, 2020). The Cyclin Dependent Kinase B1 (CDKB1) subgroup of cell-cycle markers functions from the onset of the S-phase until mitosis and regulates cell division in several plants including *Antirrhinum* (cdc2c) (Fobert *et al.*, 1996), alfalfa (cdc2MsD) (Magyar *et al.*, 1997), tobacco (Sorrell *et al.*, 2001), Arabidopsis (Segers *et al.*, 1995; Menges and Murray, 2002) and barley (Gendreau *et al.*, 2008, 2012).

Cell-cycle checkpoints maintain genomic integrity in proliferating cells to prevent aberrant replication following DNA damage or stress. In Arabidopsis, WEE1 inhibits plant growth by arresting cells in the G2-phase (De Schutter *et al*., 2007), and it also has a role in endoreplication in maize endosperm and in tomato fruit (Sun *et al.*, 1999; Gonzalez *et al.*, 2004, 2007). Members of the *Poaceae* family such as maize, barley, and wheat show a lower endoreduplication index in the leaf in comparison to *Brassicaceae* and *Cucurbitaceae* family members (Barow and Meister, 2003).

Barley is a host for numerous pathogens and insect pests that attack the plant at different growth stages. *Blumeria hordei* causes barley powdery mildew and results in considerable loss in productivity despite fungicide treatment (Kusch and Panstruga, 2017; Stam *et al*., 2017, Preprint; Cowger *et al*., 2018; Liu *et al*., 2019). Powdery mildews belong to the *Helotiales* (Johnston *et al.*, 2019), an ascomycete family representing an ancient lineage that evolved over 100 million years ago and that has diversified to more than 400 species colonizing nearly 10 000 plant species (Takamatsu, 2004; Johnson *et al*., 2010; Kusch and Panstruga, 2017). *Blumeria hordei* is an obligate biotroph, and its molecular and genetic interactions have been extensively studied (Kuhn *et al.*, 2016).

The main aim of this study was to gain insights into the spatiotemporal regulation of the responses to hormones and to *B. hordei* infection along the barley leaf. To this end, we carried out flow cytometry to monitor cell-cycle stages and ploidy, microscopy to quantify hyphae formation, and qRT-PCR to profile spatiotemporal expression patterns of different categories of genes associated with JA, GA, defence, and the cell cycle. We found that different parts of the leaf possess different growth capacities and intrinsic abilities to respond to hormones and *B. hordei* infection, and that these are further fine-tuned by MeJA and GA.

## Materials and methods

### Plant cultivation and pathogen maintenance

The barley (*Hordeum vulgare*) cultivars Golden Promise and Haruna Nijo were grown in 9 cm pots in soil (John Innes No.1) with 15–20 seeds per pot. Golden Promise was also grown in hydroponic conditions in magenta vessels (Sigma GA-7 V8505) containing 100 ml of sterile perlite and 50–60 ml of Half-strength Hoagland solution No. 2 (Sigma H2395), with nine seeds per vessel. All the barley seedlings were grown under a 16/8 h photoperiod (~140–170 μmol m^–2^ s^–1^) at 22 °C and relative humidity of ~60–70%. *Blumeria hordei* strain DH14 (provided by Dr L. Bindschedler, RHUL) was maintained on Golden Promise seedlings that were inoculated at 7 days after sowing (DAS) and grown in soil under the same conditions

### Measurement of nuclei of infected leaf epidermal cells

Primary leaves of plants were inoculated with *B. hordei* at 5 DAS (see below), and then at 7 DAS the leaves of infected and equivalent uninfected plants were used for DAPI staining (adapted from Chazotte, 2011). Microscopy observations and disease scoring in the infected leaves were according to an adapted protocol outlined by Lambertucci *et al*. (2019). The infected leaf samples were cleared with ethanol (80% v/v) and acetic acid (20% v/v) solution overnight at 4 °C in the dark, and then rinsed with phosphate buffered saline (PBS) for 30 mins. DAPI solution (2 ng µl^–1^) was added to leaf tissues for 20 min and then they were de-stained for 30 min with PBS. The DAPI-stained leaves were also stained with 2 μg ml^–1^ of propidium iodide (PI ; adapted from Scheler *et al.*, 2016). The leaves with their adaxial side up were placed on glass slides with PBS and sealed with nail varnish. Nuclei were imaged using a Nikon Eclipse Ni-E Upright microscope with preselected filters for DAPI (358/461 nm) and TxRed (535/617 nm) with a Nikon Intensilight C-HGF1 UV light source. i-stack images were taken of uninfected epidermal cells and infected nuclei (near the haustoria) and analysed using ImageJ software.

### Flow cytometry analysis

Ploidy levels were measured by flow cytometry (Sysmex CyFlow^®^ Space) using a Cystain UV Precise P high-resolution DNA staining kit (Partec, 05-5002) according to the manufacturer’s instructions. The protocol for barley sample preparation was adapted from Doležel *et al*. (2007) and Noir *et al.* (2013): fine-cutting of the tissue was carried out in Cystain UV Precise P Nuclei Extraction Buffer (Sysmex), staining with DAPI was conducted using Cystain UV Precise P Staining Buffer (Sysmex), and debris was filtered using a 20–30 µm filter (CellTrics, Sysmex) before running the sample through the flow cytometer. To identify the nuclear DNA content in developing barley, untreated Golden Promise seedlings were collected at 7 DAS and the sheath was cut into 5–10 mm sections while the proximal and distal portions of the leaf blade were cut into 20 mm sections. Golden Promise seedlings grown in soil were collected 7 DAS, infected with *B. hordei*, and then sampled at 3 days post-inoculation (DPI) and 5 DPI. The proximal and distal leaf-blade sections of these seedlings were then subjected to flow cytometry. The analysis was performed to measure the DNA content from at least 15 000 nuclei per sample based on relative fluorescence intensities, and recorded using the Sysmex FloMax software. The flow cytometry experiments were repeated for three biological replicates, with two technical replicates for each. Cell-cycle analysis was performed in parallel with the ploidy measurements, by fitting frequency histogram outcomes into the cell-cycle analysis tool to obtain the frequency of nuclei in the G0/G1, S, and G2/M phases of the cell cycle.

### Characterization of *B. hordei* infection along the primary barley leaf blade

The infection protocol was adapted from Haugaard and Collinge (2001), Achuo *et al.* (2004), Nottensteiner *et al.* (2018), and Lambertucci *et al*. (2019). The primary leaf blade of soil-grown Golden Promise at 7 DAS was detached and placed with the adaxial surface upwards on 0.6% agar (Melford P1003) containing 20 ng ml^–1^ benzimidazole (Sigma 194123) in 12 cm plates. Hydroponically grown Golden Promise seedlings were also collected 7 DAS, and their primary leaves were detached and placed on similar plates. In addition, whole seedlings of hydroponically grown Golden Promise at 7 DAS were also placed on similar plates. Spores of *B. hordei* from fresh inoculum previously propagated on Golden Promise for 7 d were delivered by shaking to the horizontally placed leaves on the plates as described by Nottensteiner *et al.* (2018). Characterization and quantification of *B. hordei* infection in the primary leaves was followed as described by Peterhansel *et al*. (1997) and Li *et al*. (2020), with a spore density of 15–40 mm^–2^ in a sealed chamber and the infection was allowed to develop under the plant growth conditions described above. Conidia, appressoria, and hyphae formation in the distal and proximal leaf-blade sections were observed and counted using a light microscope. The data were converted to percentages of hyphae formed relative to the total number of ungerminated conidia, appressoria, and hyphae-forming conidia.

### Phytohormone treatments

Either 0.1 µM or 10 µM GA_3_ (Sigma) was added to the medium of the hydroponically grown Golden Promise seedlings from seed germination until 7 DAS (concentrations based on physiological responses determined by Uçarli, 2021). Similarly, 50 μM MeJA was applied to the hydroponically grown barley seedlings from 4 DAS to 7 DAS (concentration based on Noir *et al.*, 2013; Bömer *et al.*, 2018). Sampling of the treated seedlings was performed 7 DAS, when the sheaths were dissected into segments that were 0–0.5 cm, (A) 0.5–1.5 cm (B), 1.5–2.5 cm (C), and 2.5–3.5 cm (D) from the seed. Similarly, the leaf blade was dissected into 2 cm sections in the proximal and distal portions. The samples were used for flow cytometry analysis of nuclear DNA content and cell-cycle stages.

Another batch of Golden Promise seedlings grown in hydroponic conditions was treated with either 10 µM GA_3_ as described above, or with 50 μM MeJA for 24 h (from 5 DAS to 6 DAS) after which they were transferred to fresh half-strength Hoagland solution. At 7 DAS, leaves were cut from the seedlings and infected with *B. hordei* as described above. Proximal and distal leaf-blade samples were taken at 5 DPI for assessment of the spread of infection.

A third batch of Golden Promise seedlings were grown hydroponically and treated as described above with either 10 μM GA_3_ for 7 d or 50 μM MeJA for 24 h, and were then infected with *B. hordei*. Proximal and distal leaf-blade samples were collected 2, 3, and 5 DPI for molecular analysis.

### Gene expression analysis by quantitative real-time PCR

For gene expression analysis, the sheath and leaf-blade samples from three seedlings per treatment were each pooled to form a biological replicate. Total RNA was isolated using RNeasy Plant Mini Kit (Qiagen) following the manufacturer’s protocol. cDNA preparation was performed using a QuantiTect Reverse Transcription Kit (Qiagen). Real-time amplification was performed using SYBR Green JumpStart (Sigma-Aldrich) according to the manufacturer’s instructions. Transcript analysis was performed using RNA samples derived from at least three independent biological replicates. The comparative 2^–ΔΔ*C*T^ method (Livak and Schmittgen, 2001; Pfaffl, 2001; Vandesompele *et al.*, 2002; Noir *et al.*, 2013) was used to evaluate the transcript abundance of each gene in the samples relative to the reference genes *Ubiquitin* and *GAPDH*. Primer sequences are listed in Supplementary Table S1.

## Statistical analysis

One-way ANOVA was used to identify significant differences in the molecular and flow-cytometry experiments. One-way ANOVA followed by Tukey’s post-hoc test was used in the experiments involving *B. hordei* infection in the leaf blade with or without phytohormone treatment, and for assessment of nuclear enlargement in epidermal cells following *B. hordei* infection. Statistical analysis was performed using Graphpad Prism as well as the Xrealstats analysis tool in Microsoft Excel.

## Results

### MeJA and GA treatments differentially regulate the cell cycle in barley sheath and leaf blade

**Fig. 1.**
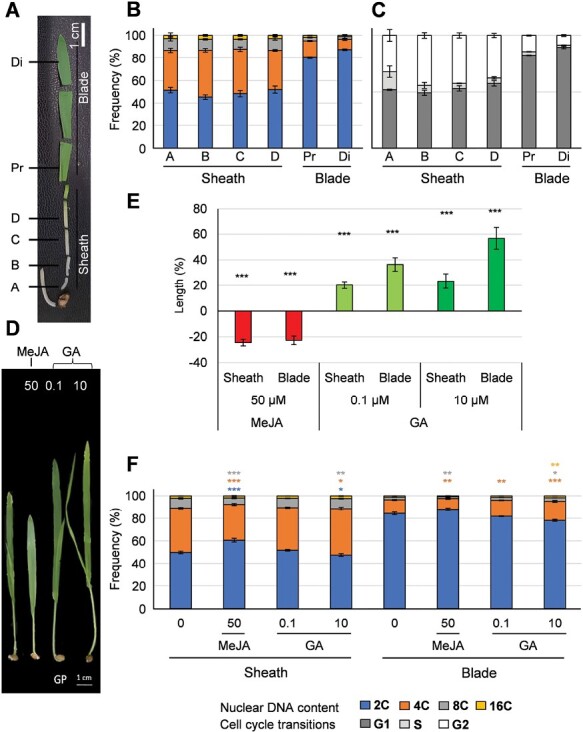
Frequencies of different nuclear DNA content and cell-cycle stages in the sheath and leaf blade of barley seedlings, and effects of MeJA and GA treatments. (A–C) Seedlings of the cultivar Golden Promise were grown hydroponically and sampled at 7 days after sowing (DAS). Di, distal; Pr, proximal. (A) Samples that were taken from different sections of the sheath and leaf blade. (B) Frequencies of nuclear DNA content (ploidy levels) in the different sections. (C) Frequencies of cells in different stages of the cell cycle. (D–F) Seedlings of Golden Promise (GP) were grown hydroponically. For the JA treatment, 50 µM MeJA was added to the solution at 4 DAS. For the GA treatment, either 0.1 µM GA_3_ or 10 µM GA_3_ was present in the solution from the time of sowing. Samples of whole sheaths and whole leaves were taken at 7 DAS. (D) Representative images of control and treated seedlings at 7 DAS. (E) Relative length of the sheath and leaf blade as a percentage of the control. Data are means (±SE), of a minimum of three biological replicates. (F) Frequencies of nuclear DNA contents in the different treatments. Data in (B, C, F) are means (±SE) from 15 000–20 000 nuclei counted from three biological replicates, each of which consisted of 3–4 pooled seedlings. Significant differences compared with the untreated control in (E, F) were determined using one-way ANOVA: *P<0.05, **P<0.01, ***P<0.001.

In monocotyledon leaves, the sheath and the leaf blade undergo distinct developmental processes, with the sheath emerging last from the meristem (Ramírez-González *et al.*, 2018). These two primary leaf portions from the barley cultivar Golden Promise were separated and dissected at 7 DAS), and the cell cycle stages along their length axes were examined using flow cytometry (Fig. 1A). The frequency of nuclei with 4–16 C ploidy was higher in the sheath than in the proximal and distal portions of the primary leaf blade (Fig. 1B), and this is consistent with a relatively higher mitotic activity in the sheath, where a higher frequency of cells in the S/G2-M phase of the cell cycle was observed compared to the leaf blade (Fig. 1C; Table 1; Supplementary Table S2). Similarly, higher ploidy levels were observed in the sheath of the fast-growing barley cultivar Haruna Nijo (Groszyk and Szechynska-Hebda, 2021; Supplementary Fig. S1), suggesting that cell ploidy and cell cycle transition stages in barley are probably independent of the growth rate of the cultivar.

**Table 1.** Frequencies of nuclei exhibiting 2C–16C DNA content and cell-cycle transition states in barley sheath and leaf blade, and effects of MeJA and GA treatments

	Sheath				Leaf blade		Sheath				Leaf blade			
	A	B	C	D	Pr	Di	Control	JA(50)	GA(0.1)	GA(10)	Control	JA(50)	GA(0.1)	GA(10)
Ploidy														
2C	51.44 ± 2.38	45.32 ± 1.98	49.23 ± 2.83	52.68 ± 3.20	80.17 ± 0.81	88.64 ± 0.76	45.39 ± 1.1	54.58 ± 1.5	47.34 ± 0.7	43.63 ± 1.1	78.92 ± 1.1	78.46 ± 1.1	78.77 ± 0.3	74.88 ± 0.8
4C	35.30 ± 1.91	41.52 ± 1.59	40.00 ± 1.93	35.34 ± 0.79	14.91 ± 0.46	9.26 ± 0.91	35.81 ± 0.6	28.64 ± 0.9	34.45 ± 0.6	37.74 ± 1.2	11.29 ± 0.4	8.57 ± 0.7	13.79 ± 0.3	16.33 ± 0.8
8C	10.34 ± 2.01	9.70 ± 0.61	9.01 ± 0.51	10.54 ± 1.33	3.14 ± 0.43	2.52 ± 1.75	8.02 ± 0.7	5.21 ± 0.5	7.65 ± 0.2	8.20 ± 0.4	2.27 ± 0.10	1.56 ± 0.2	2.55 ± 0.1	2.98 ± 0.1
16C	2.91 ± 2.20	3.44 ± 0.80	3.48 ± 0.81	2.87 ± 0.79	1.77 ± 0.18	1.00 ± 0.22	1.95 ± 0.1	1.65 ± 0.3	1.95 ± 0.14	2.22 ± 0.3	1.08 ± 0.10	0.55 ± 0.2	1.25 ± 0.1	1.63 ± 0.1
Cell cycle														
G1	51.89 ± 0.58	49.27 ± 2.31	53.06 ± 2.32	57.58 ± 2.37	82.52 ± 0.26	89.41 ± 1.08								
S	15.93 ± 5.01	6.42 ± 2.78	4.51 ± 0.32	4.73 ± 1.11	3.17 ± 0.28	1.93 ± 0.39								
G2	32.17 ± 5.21	44.30 ± 2.44	42.42 ± 2.28	37.68 ± 1.86	14.30 ± 0.48	8.64 ± 0.78								

The sheath and leaf-blade sections are illustrated in Fig. 1A, and the data correspond to Fig. 1B: Pr, proximal; Di, distal. Data for whole sheaths and leaves correspond to Fig. 1F: JA(50), 50 µM MeJA; GA(0.1), 0.1 µM GA_3_; GA(10), 10 µM GA_3_ (see Fig. 1 for details). The analyses were performed on at least 15 000 nuclei isolated from three sheath/leaves for each ploidy measurement. Cell cycle analysis was performed in parallel to the ploidy measurements (see Methods).

The phytohormones JA and GA play different roles in growth, development, and nuclear DNA distribution in the leaf (Maucher *et al*., 2000; Chandler *et al*., 2008; Noir *et al*., 2013; Kanno *et al*., 2016; Patil *et al.*, 2019; Pérez-Salamó *et al*., 2019). To determine their effects on the growth of the sheath and leaf blade, we applied either 50 µM MeJA, 0.1 µM GA_3_, or 10 µM GA_3_ (effective concentrations to induce physiological responses based on Noir *et al*., 2013, and Bömer *et al*., 2018). The MeJA treatment significantly reduced the lengths of both the sheath and leaf blade by ~20% (*P*=7.82 × 10^–7^ and *P*=3.0 × 10^–15^, respectively) when compared to the untreated controls (Fig. 1D, E). In contrast, both the GA treatments increased sheath length by ~20% and also promoted leaf blade elongation by 36% at 0.1 µM GA_3_ (*P*=5.0 × 10^–4^), and by 56% at 10 µM GA_3_ (*P*=1.05 × 10^–5^).

Flow cytometry was used to determine the effects of JA and GA on the progression of the cell cycle in the sheaths and leaf blades. Compared with the control, the MeJA treatment significantly increased the frequency of 2C nuclei (*P*=2.0 × 10^–6^) and reduced the frequency of 4C (*P*=2.6 × 10^–8^) and 8C (*P*=5.0 × 10^–4^) nuclei in the sheath, while smaller differences were observed in the leaf blade (Fig. 1F; Table 1). In the leaf blade, the MeJA treatment significantly reduced the frequency of 4C (*P*=4.3 × 10^–3^) and 8C (*P*=9.1 × 10^–3^) nuclei compared with the untreated control. The 0.1 µM GA treatment did not show a significant effect on the nuclear DNA content in the sheath; however, the 10 µM GA treatment resulted in significant increases in 4C (*P*=0.035) and 8C (*P*=9.6 × 10^–3^) nuclei, and a corresponding decrease in 2C nuclei (*P*=0.026). In the leaf blade, there were significant dose-dependent increases in the frequency of 4C nuclei at both GA concentrations, and a significant increase in 8C nuclei at 10 µM GA. These results suggested that GA and JA have opposite effects on the cell cycle and growth in the barley leaf, with GA causing increases in elongation and nuclear DNA content (ploidy), whilst JA reduces them.

### MeJA and GA treatments differentially regulate the expression of cell-cycle marker genes in the barley leaf

**Fig. 2.**
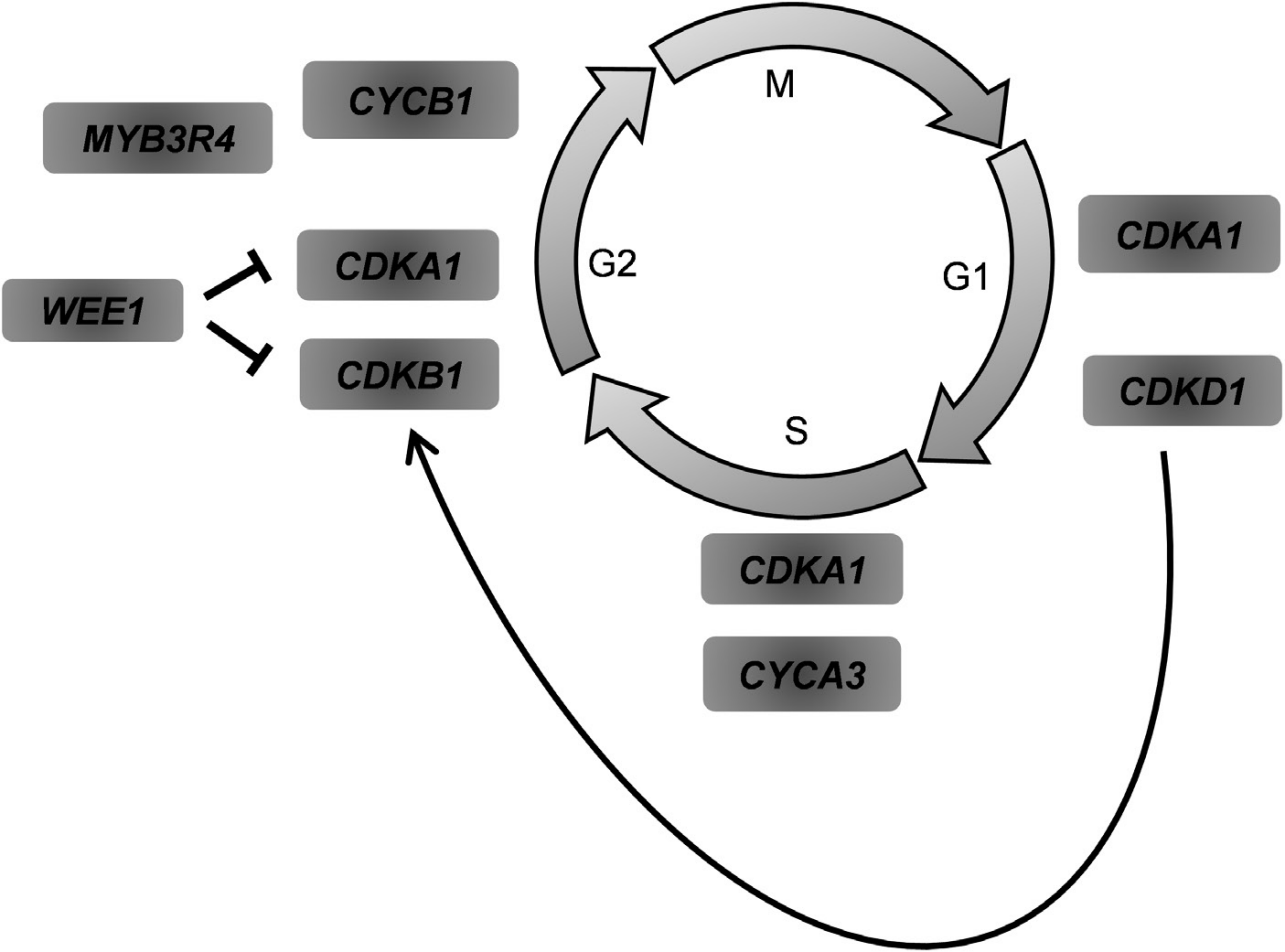
Overview of the marker genes for the cell cycle and cell proliferation analysed in this study. *CDKA1*, *Cyclin-Dependent Kinase A1*; *CDKD1*, *Cyclin-Dependent Kinase D1*; *CDKB1*, *Cyclin-Dependent Kinase B1*; *CYCB1*, *Cyclin B1*. See text for details.

**Fig. 3.**
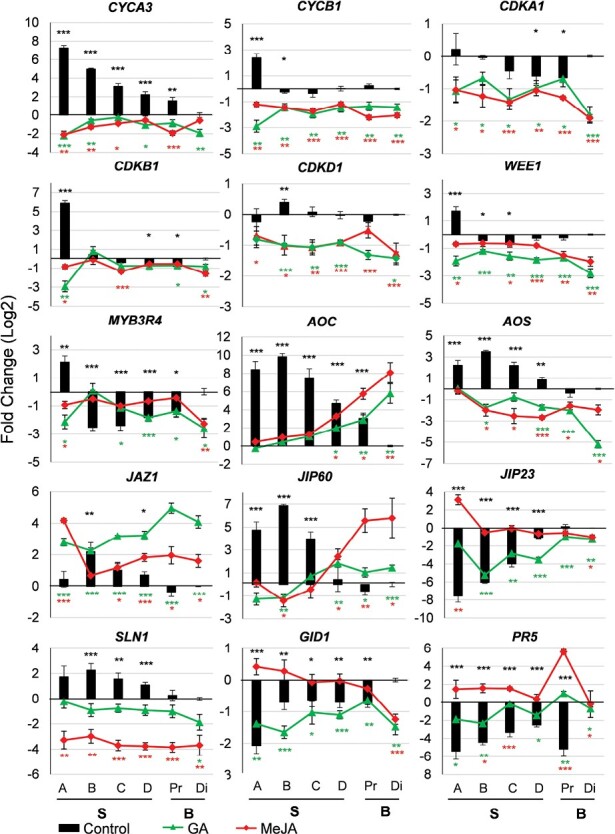
Expression of genes related to the cell cycle, JA, GA, and plant defence in the sheaths and leaf blades of barley seedlings in response to JA and GA treatment. Seedlings of the cultivar Golden Promise were grown hydroponically. For the JA treatment, 50 µM MeJA was added to the solution at 4 days after sowing (DAS). For the GA treatment, either 0.1 µM GA_3_ or 10 µM GA_3_ was present in the solution from the time of germination. Samples of different sections of the sheath (S) and leaf blade (B) were taken at 7 DAS, as illustrated in Fig. 1A. Pr, proximal; Di, distal. Expression was determined by qRT-PCR relative to the reference genes *Ubiquitin* and *GAPDH*. The values for the untreated leaf and sheath controls (black bars) are shown as the log_2_ fold-change relative to the expression in the distal blade; the results for MeJA and GA (red and green lines respectively) are the log_2_ fold-change relative to the corresponding control value. Data are means (±SE) of three biological replicates (*n*=3 experiments, with 3–4 seedlings per experiment, with at least three technical replicates). One-way ANOVA was used to determine significant differences compared with the distal leaf section for the control values, and compared with the corresponding control value for the MeJA and GA treatments: *P<0.05, **P<0.01, ***P<0.001. Genes associated with the cell cycle: *CYCA3*, *CYCB1*, *CDKA1*, *CDKB1*, *CDKD1*, *WEE1*, and *MYB3R4*. Genes associated with GA and JA biosynthesis and signalling: *AOC*, *AOS*, *JAZ1*, *JIP60*, *JIP23*, *SLN1*, and *GID1*. Genes associated with defence: *PR5*.

To gain insights into the molecular mechanisms regulating the barley response to the hormone treatments, we next used qRT-PCR to examine the expression of signature genes associated with the cell cycle and proliferation along the sheath and leaf blade. The genes have previously been characterized by Gendreau *et al*. (2008, 2012), included the following: *Cyclin A3* (*CYCA3*, *HORVU.MOREX.r2.5HG0363660*; a checkpoint regulator of S, S/G2 transition; Gendreau *et al*., 2012; Qi and Zhang, 2020); *Cyclin B1* (*CYCB1*, *HORVU.MOREX.r2.3HG0246040*; a checkpoint regulator at the G2/M transition; Dewitte *et al.*, 2003); *Cyclin-Dependent Kinase A1 (CDKA1*, *HORVU.MOREX.r2.6HG0457700*; known to be constitutively expressed throughout the cell cycle and acts as a positive regulator of cell proliferation; Iwakawa *et al*., 2006; Gendreau *et al*., 2012), *Cyclin-Dependent Kinase B1* (*CDKB1*, *HORVU.MOREX.r2.7HG0565000*; functions during G2/M and the S/G2 phase of the cell cycle; Dewitte *et al.*, 2003; Gendreau *et al*., 2012); *Cyclin-Dependent Kinase D1* (*CDKD1*, *HORVU.MOREX.r2.7HG0565000*; its expression increases from G1 to S phase, and it mediates cell-cycle progression through the activation of *CDK*s such as *CDKA1* and *CDKB1*; Gendreau *et al*., 2012; Gutierrez *et al.*, 2009; Takatsuka *et al*., 2015); and the nuclear kinase *WEE1* (*HORVU.MOREX.r2.6HG0461230*; a negative regulator of the G2/M phase that inhibits the activities of *CDKA* and *CDKB*; De Schutter *et al.*, 2007; Qi and Zhang, 2020). The *MYB3R4* transcription factor (*HORVU.MOREX.r2.3HG0206530*) has not been formally identified as a cell-cycle marker, but it controls the expression of G2/M phase-specific genes and maintains diploidy (Saito *et al.*, 2015; Haga *et al.*, 2007, 2011). For a schematic depiction of the known roles of these genes in the cell cycle, see Fig. 2. Using the distal leaf-blade segment as the reference, the overall expression of these cell cycle-related genes under control conditions was higher in the sheath than in the blade, especially at its base, the region closest to the meristem and more actively dividing (Fig. 3).

Compared to the control, treatment with 10 µM GA and 50 µM MeJA both significantly reduced the expression of these genes in the sheath and blade tissues (Fig. 3). These results supported a role, either direct or indirect, of JA and GA in modulating leaf growth by acting on common cell-cycle regulators.

### MeJA and GA treatments modify the expression of genes associated with hormones and defence in the leaf

To understand the effects of exogenous GA and JA on defence-related genes in the barley sheath and leaf blade, transcription profiling was performed for *Allene Oxide Synthase* (*AOS*, *HORVU.MOREX.r2.4HG0328610*) and *Allene Oxide Cyclase* (*AOC*, *HORVU.MOREX.r2.6HG0514200*), which are responsible for the first steps of the JA biosynthesis pathway in the chloroplasts and have been shown to be modulated by exogenous JAs and pathogen attack (Maucher *et al.*, 2000, 2004). In addition, to gain insights into the relationships between phytohormone signalling and changes in cell-cycle progression and elongation in the sheath and leaf blade, the expression of other JA-associated genes including the JA-signalling repressor *Jasmonate-Zim-Domain1* (*JAZ1*, *HORVU.MOREX.r2.2HG0103320*; Qi *et al*., 2011), *Jasmonate Inducible Protein 60 kDa* (*JIP60*, *HORVU.MOREX.r2.4HG0345940*; a ribosome-inactivating protein; Chaudhry *et al*., 1994; Rustgi *et al*., 2014), and *Slender 1* (*SLN1*, *HORVU.MOREX.r2.4HG0280720*; encoding a DELLA protein that modulates the response between JAs and GAs and represses elongation; de Vleesschauwer *et al*., 2016), was also analysed. The analysis showed that the basal expression of *AOC* (section A, *P*=3.6 × 10^–6^; section B, *P*=3.4 × 10^–9^; section C, *P*=3.3 × 10^–5^; section D, *P*=2.4 × 10^–7^), *AOS* (section A, *P*=7.0 × 10^–4^; section B, *P*=6.8 × 10^–10^; section C, *P*=2.7 × 10^–5^; section D, *P*=7.3 × 10^–3^), *JAZ1* (section B, *P*=4.0 × 10^–3^), *JIP60* (section A, *P*=6.9 × 10^–5^; section B, *P*=3.4 × 10^–9^; section C, *P*=1.0 × 10^–4^) and *SLN1* (section B, *P*=7.0 × 10^–4^; section C, *P*=8.7 × 10^–3^; section D, *P*=7.0 × 10^–4^) was higher in the sheath compared to the distal leaf blade in the untreated control (Fig. 3). Treatment with 10 µM GA and with 50 µM MeJA induced expression of *AOC*, *JAZ1*, and *JIP60* in the distal leaf blade compared with the control. Unlike the other JA-responsive genes, the expression of *Jasmonate Inducible Protein 23 kDa* (*JIP23*, *HORVU.MOREX.r2.6HG0512860*; possibly involved in sugar transport and seed development; Oikawa *et al*., 2007) was higher in the leaf blade compared to the sheath in the control.

Treatment with either MeJA or GA significantly reduced *AOS* expression on average by 2–2.5-fold in the sheath and leaf blade compared with the control (Fig. 3), Expression of *SLN1* (encoding a DELLA protein) was also reduced by GA and MeJA treatment in the sheath by ~1.5- and 3-fold, respectively, (section A, *P*=5.05 × 10^–3^; section B, *P*=2.26 × 10^–3^; section C, *P*=1.89 × 10^–4^; section D, *P*=1.33 × 10^–5^)and in the leaf blade by ~2-fold (GA in distal section, *P*=2.84 × 10^–2^) and 4-fold (MeJA in proximal section, *P*=9.68 × 10^–5^; distal, *P*=9.35 × 10^–3^), respectively. GA treatment reduced *JIP23* expression significantly compared with the control in the sheath (section B, *P*=2.46 × 10^–4^; section C, *P*=1.27 × 10^–3^; section D, *P*=3.97 × 10^–6^), while MeJA treatment increased it by 3-fold in section A (*P*=2.2 × 10^–3^, i.e. more proximal to the meristem). *AOC* expression was induced by both GA and JA in the blade and decreased in the sheath. The expression patterns observed for *AOC* and *AOS* were in agreement with previous studies on barley (Maucher *et al.*, 2000, 2004).

In the controls, the expression of the gibberellin receptor *Gibberellin-Insensitive Dwarf1* (*GID1*, *HORVU.MOREX.r2.1HG0049800*; regulating growth and development; Ueguchi-Tanaka *et al*., 2005) and the defence-related gene *Pathogenesis Related 5* [*PR5*, *HORVU.MOREX.r2.5HG0351950*; belonging to the thaumatin-like protein family, suggested to interact with (1,3)-β- d-glucans commonly found in fungal walls; Osmond *et al*., 2001] were broadly comparable to *JIP23* in the sheath (Fig. 3). The expression levels of *GID1*, *PR5*, and *JIP23* in the controls were particularly low in all segments of the sheath. Notably, *GID1* expression was reduced by ~1.5-fold in the distal leaf blade by both the GA and JA treatments compared to the control (GA, *P*=1.84 × 10^–3^, MeJA, *P*=2.81 × 10^–4^), while that of *PR5* was 1.5-fold higher in the sheath and ~5-fold in the proximal section (*P*=3.06 × 10^–7^) of the leaf blade following MeJA treatment. Taken together, these results showed the existence of expression signatures associated with the sheath and leaf blade that could be linked to cell’s distinct abilities to respond to hormone-induced stress.

### The proximal and distal sections of the leaf blade show different susceptibility to *B. hordei* infection

**Fig. 4.**
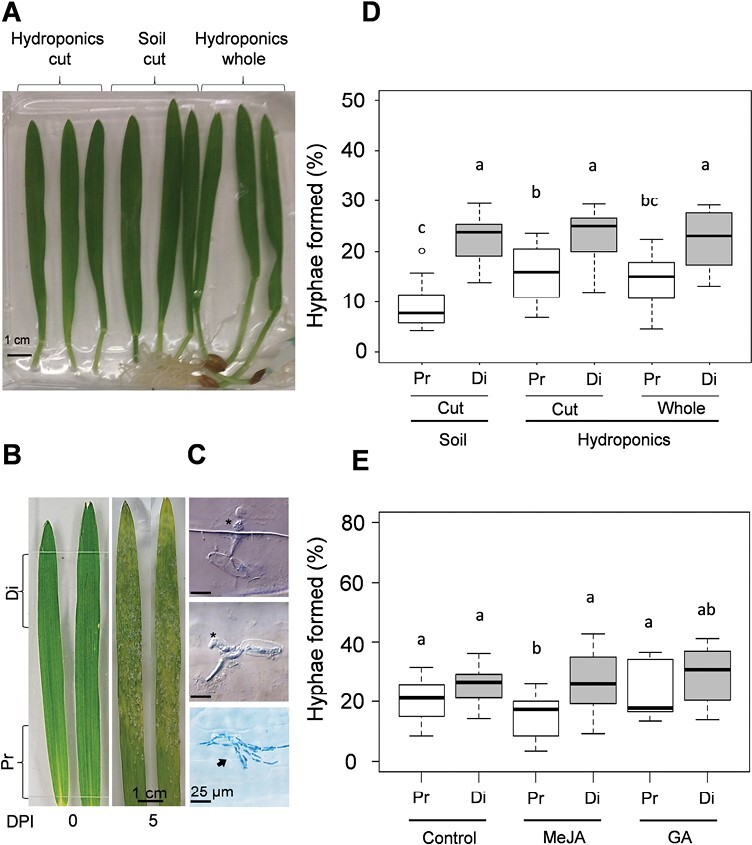
Effects of barley culture conditions on formation of *Blumeria hordei* hyphae on the leaf blade, and effects of pre-treatment with MeJA on *B. hordei* infection. (A) Barley seedlings were grown either in soil or hydroponically for 7 days after sowing (DAS), after which detached leaves from both were infected with *B. hordei* (15–40 spores mm^–2^), and attached leaves on hydroponically grown seedlings were also infected. (B) Representative images of detached blades of hydroponically grown plants at 0 days post-inoculation (DPI) and 5 DPI. Di, distal blade section; Pr, proximal blade section. (C) Microscopic images of *B. hordei* structures at 2DPI at 2 DPI on the leaf blade. Asterisks indicate appressoria (top two panels) and the arrow indicates hyphae formation (bottom panel). (D) Hyphae formation on the proximal and distal sections of the leaf blades shown in (A), expressed as the percentage of hyphae formed relative to the total number of ungerminated conidia, appressoria, and hyphae-forming conidia at 2 DPI. Data are means of at least three biological replicates. (E) Seedlings of the cultivar Golden Promise were grown hydroponically. For the MeJA treatment, 50 µM MeJA was added to the solution for 24 h at 5–6 DAS, and then the medium was substituted with half-strength Hoagland’s solution No 2. Samples were taken at 7 DAS. For the GA treatment, 10 M GA_3_ was present in the solution from the time of germination. At 7 DAS, leaves were detached and infected with *B. hordei*, and hyphae formation in the proximal and distal sections was assessed at 2 DPI. (D, E) *n*=6 experiments with at least three leaves per experiment. Different letters indicate significant differences among means as determined using one-way ANOVA and Tukey’s post-hoc test (*P*<0.05).

Barley seedlings were cultivated either in soil or hydroponic conditions and then detached leaves or whole seedlings were infected with *B. hordei* in order to evaluate the distribution of infection along the leaf blade and to test whether the process was affected by the excision of the leaf from the plant (Fig. 4A). Infection at the macroscopic scale was clear at 5 DPI, with colony clusters more abundant in the distal section (Fig. 4B). Hyphae formation from the germinated conidia was quantified 2 DPI, when it was possible to distinguish individual colonies according to (Li *et al.*, 2020) (Fig. 4C). Irrespective of growth conditions or cutting, the hyphae formation was greater on the distal leaf blade (Fig. 4D), mimicking the natural process of infection. Compared to the proximal leaf blade of cut leaves from hydroponic seedlings, ~58% (*P*=4.0 × 10^–2^) more hyphae were observed in the distal leaf blade. The hyphae formation in hydroponically grown barley seedlings was ~30% lower in the proximal leaf section whether the leaf was infected as part of the whole seedling (*P*=1.9 × 10^–3^) or detached (*P*=4.2 × 10^–2^).

To examine their effects on *B. hordei* infection, seedlings were pre-treated with either 50 μM MeJA or 10 μM GA. Compared with the control, MeJA treatment reduced hyphae formation (*P*=8.0 × 10^–4^) in the proximal leaf blade by an average of ~33% but had no detectable effect in the distal leaf blade (Fig. 4E). In contrast, the GA pre-treatment had a positive effect on hyphae formation in the leaf.

The distribution pattern of *B. hordei* infection along the leaf blade therefore reflected the gradient of JA- and GA-related gene expression along the leaf (Figs 3, 4D) and it was not affected by wounding; instead it reflected the existence of an intrinsic regulatory system that was possibly modulated by JA and GA to protect the cells near the meristem from pathogen attack.

### Genes associated with the cell cycle, hormones, and defence show different expression patterns in the leaf blade following *B. hordei* infection

**Fig. 5.**
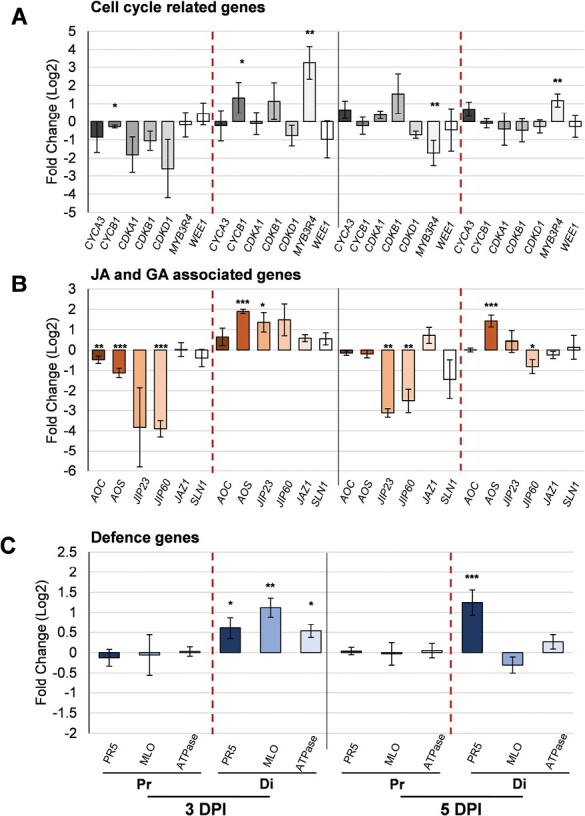
Effects of *Blumeria hordei* infection on the expression of genes associated with the cell cycle, JAs, GA, and plant defence in barley leaf blades. Detached leaves from seedlings at 7 days after sowing (DAS) were infected with *B. hordei* and samples from the proximal (Pr) and distal (Di) sections were collected for analysis at 3 d post-inoculation (DPI) and 5 DPI. Expression was determined using qRT-PCR with *GAPDH* as the reference gene. For each gene analysed, the results are presented as the fold-change in log_2_ expression in the infected sample compared to the corresponding control sample. (A) Genes associated with the cell cycle, (B) genes associated with JAs and GA, and (C) genes associated with plant defence. Data are means (±SE) of four biological replicates, each consisting of samples pooled from three leaves. Significant differences in the fold-change in expression were determined using one-way ANOVA: *P<0.05, **P<0.01, ***P<0.001.

To understand of the spatiotemporal effect of hormones on *B. hordei* infection of barley leaf, the expression of genes related to the cell cycle, JA, and defence in uninfected and *B. hordei-*infected, proximal and distal leaf blades at 3 DPI and 5 DPI was next examined. The same set of cell-cycle marker genes described in Fig. 3 was analysed to assess whether the pathogen altered their expression and interfered with cell proliferation *in planta*. Relative to the uninfected control, the expression of the two G2/M phase marker genes *CYCB1* and *CDKB1* increased by ~1.3-fold in infected distal leaf blades at 3 DPI, although it was only significant for *CYCB1* (*P*=2.06 × 10^–2^; Fig. 5A). Expression of the diploidy maintenance transcription factor *MYB3R4* also showed a significant 3-fold increase in the infected distal leaf blade at 3 DPI (*P*=2.1 × 10^–3^). The expression of the three genes subsequently decreased at 5 DPI, but *MYB3R4* expression remained significantly higher in the distal infected leaf blade by ~1.2-fold compared to the control (*P*=2.69 × 10^–2^). The expression of the S/SG2 marker *CYCA3* in infected distal leaf blades increased from 3 DPI (*P*=0.66) to 5 DPI(*P*=0.435), and it was higher than in the control at 5 DPI, although this was not statistically significant. The transcript levels of *CDKA1*, *CDKD1*, and *WEE1* in infected distal leaf blades either decreased or remained the same at 3 DPI and 5 DPI.

In infected proximal leaf blades, expression of *CYCA3*, *CDKA1*, and *CDKB1* was higher than that of the control at 5 DPI, but not at a significant level (Fig. 5A). The expression of *CYCA3*, *CDKA1*, *CDKB1*, and *CDKD1* increased from 3 DPI to 5 DPI. The expression of *MYB3R4* was significantly lower than in the control at 5 DPI. The overall increase in the expression of a sub-set of cell-cycle marker genes alongside the decrease in *MYB3R4* expression in the proximal leaf blade during the infection process, illustrates the ability of the fungus to induce a stress response while modulating host cell growth.

Exogenous MeJA reduced *B. hordei* infection in the proximal leaf blade (Fig. 4E) and therefore the expression of JA-biosynthesis and -signalling genes was examined to gain clues about the manipulation of the JA pathway by the pathogen during the infection process. In infected distal leaf blades the expression levels of the JA biosynthesis genes *AOC* and *AOS* were ~0.6-fold and ~2-fold higher (*P*=1.1 × 10^–16^), respectively, than in the control at 3 DPI (Fig. 5B). Similarly, the expression of the JA-inducible genes *JIP23* and *JIP60* was ~1.4-fold higher (*JIP23*, *P*=1.44 × 10^–2^). In contrast, the expression of the JA biosynthesis genes *AOS* (*P*=1.3 × 10^–5^) and *AOC* (*P*=8.2 × 10^–3^) and the JA-induced genes *JIP60* (*P*=2.6 × 10^–5^) was lowered significantly by *B. hordei* infection in the proximal leaf blade at 3 DPI compared to the uninfected control. The expression of the JA-signalling gene *JAZ1* increased slightly in the infected proximal leaf blade from 3 DPI to 5 DPI. Compared to the control at 5 DPI, *AOS* expression was 1.4-fold higher (*P*=1.3 × 10^–5^) in the infected distal leaf blade, *AOC* and *JIP23* expression remained relatively unchanged, while *JIP60* expression was significantly reduced (*P*=1.7 × 10^–2^). From 3 DPI to 5 DPI, the expression of *AOC*, *JIP23*, *JIP60*, and *JAZ1* decreased in the distal leaf blade. *SLN1* has been shown to affect node growth, GA/JA interactions, and infection (de Vleesschauwer *et al.*, 2016). In infected proximal leaf blades, *SLN1* expression was lower at 3 DPI and 5 DPI than in the control whereas in infected distal leaf blades it was higher at 3 DPI but unchanged 5 DPI. Taken together, the decrease in the overall expression of JA-related genes from 3 DPI to 5 DPI in the infected distal leaf blade might be indicative of an activated, but not maintained, host stress response at the earlier time-point.

*Mildew Locus O* (*MLO*, *HORVU.MOREX.r2.4HG0342080*) is a well-known susceptibility factor in powdery mildew infection (Devoto *et al*., 1999; Kusch and Panstruga, 2017) and *PR5* is associated with resistance against it (Lambertucci *et al*., 2019). In our study, *PR5* and *MLO* expression increased significantly by ~0.6-fold (*P*=2.03 × 10^–2^) and 1.1-fold (*P*=1.2 × 10^–3^), respectively, in infected distal leaf blades at 3 DPI relative to the control (Fig. 5C). At 5 DPI, *PR5* expression remained significantly higher than in the control (~1.3-fold; *P*=2.0 × 10^–4^), while *MLO* expression was lower, albeit not significantly (*P*=0.156). A proton pump in the plasma membrane, ATPase, regulates plant growth and JA signalling (Visnovitz *et al.*, 2012), and its expression is enhanced by bacterial infection (Zhou *et al.*, 2015). We found that *ATPase* expression was higher in the infected distal leaf blades than in the control at 3 DPI and 5 DPI (*P*=2.7 × 10^–2^). Whether the inducibility of *ATPase* was the result of *B. hordei* manipulation of the host pump to facilitate its infection remains to be determined.

### The size of nuclei increases in *B. hordei*-infected epidermal cells

**Fig. 6.**
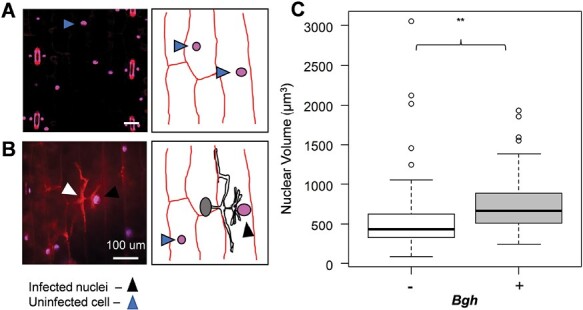
Effect of *Blumeria hordei* infection on barley epidermal cell nuclei size. Primary leaf blades of the cultivar Golden Promise were detached and infected with *B. hordei*. After 2 d, tissue samples were cleared and stained with propidium iodide (PI) and DAPI. (A, B) Representative images and corresponding diagrams of epidermal cell nuclei of (A) an uninfected leaf and (B) of a leaf infected with *B. hordei*, showing an appressorium (arrowhead). (C) Nuclear volumes of epidermal cells with (+) or without (–) infection by *B. hordei* (*Bgh*). The box-plots show results from a minimum of three biological replicates with 3–4 leaves per experiment. A total of 114 control nuclei and 83 infected nuclei were imaged. The significant difference between the infected and uninfected samples was based on a linear regression model (*P*=0.0023).

The nuclear DNA content differed between the barley leaf blade and sheath (Fig. 1B), and no significant changes were observed following infection with *B. hordei* (Supplementary Fig. S2). We used fluorescence microscopy to determine the changes in nuclear size (Chandran *et al.,* 2010, 2013; Scheler *et al.*, 2016) in infected epidermal cells adjacent to the *B. hordei* haustoria and in uninfected cells (Fig. 6A). The mean volume of the nucleus in uninfected epidermal cells was 527 µm^3^ (median 440 µm^3^) and this increased significantly by 40% in infected cells (*P*=1.0 × 10^–4^; Fig. 6B, C). The nuclear enlargement in the cells adjacent to the *B. hordei* infection site was not accompanied by any apparent increase in their fluorescence (*P*=0.803), suggesting that the increased size of the nuclei in the infected epidermal cells did not result from higher DNA content.

### Pre-treatment with MeJA or GA modifies gene expression patterns in the *B. hordei*-infected leaf blade

**Fig. 7.**
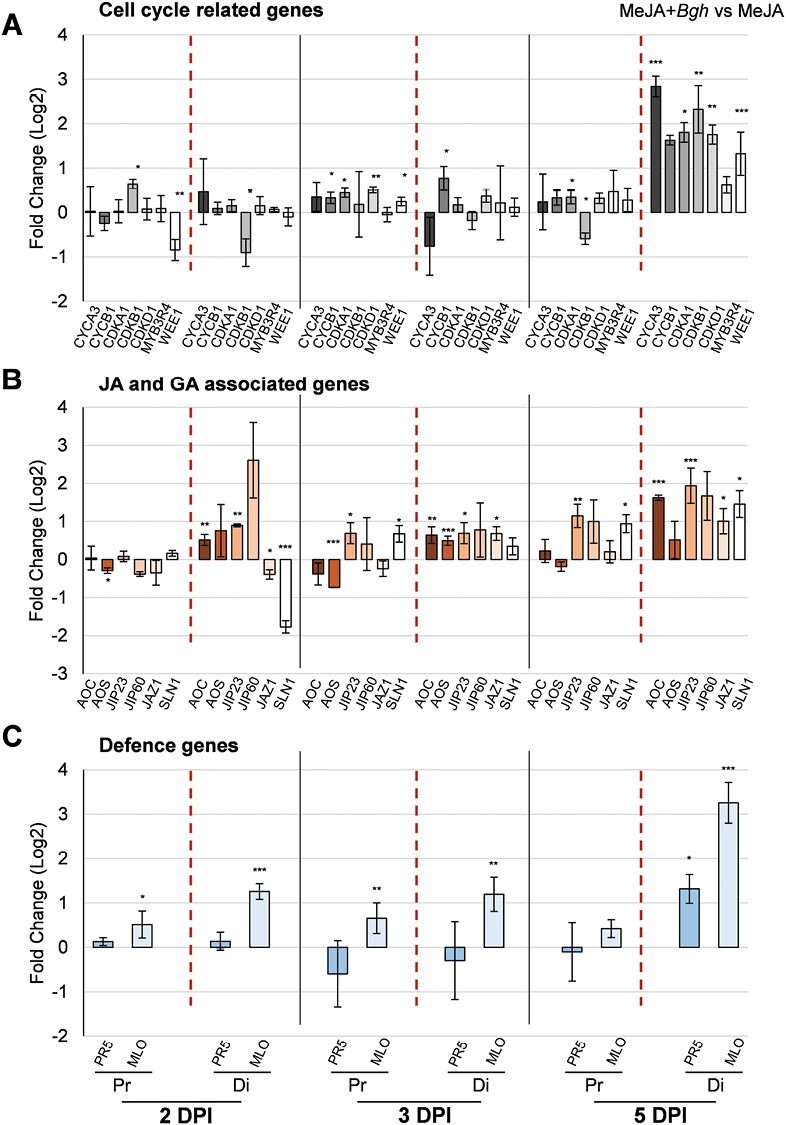

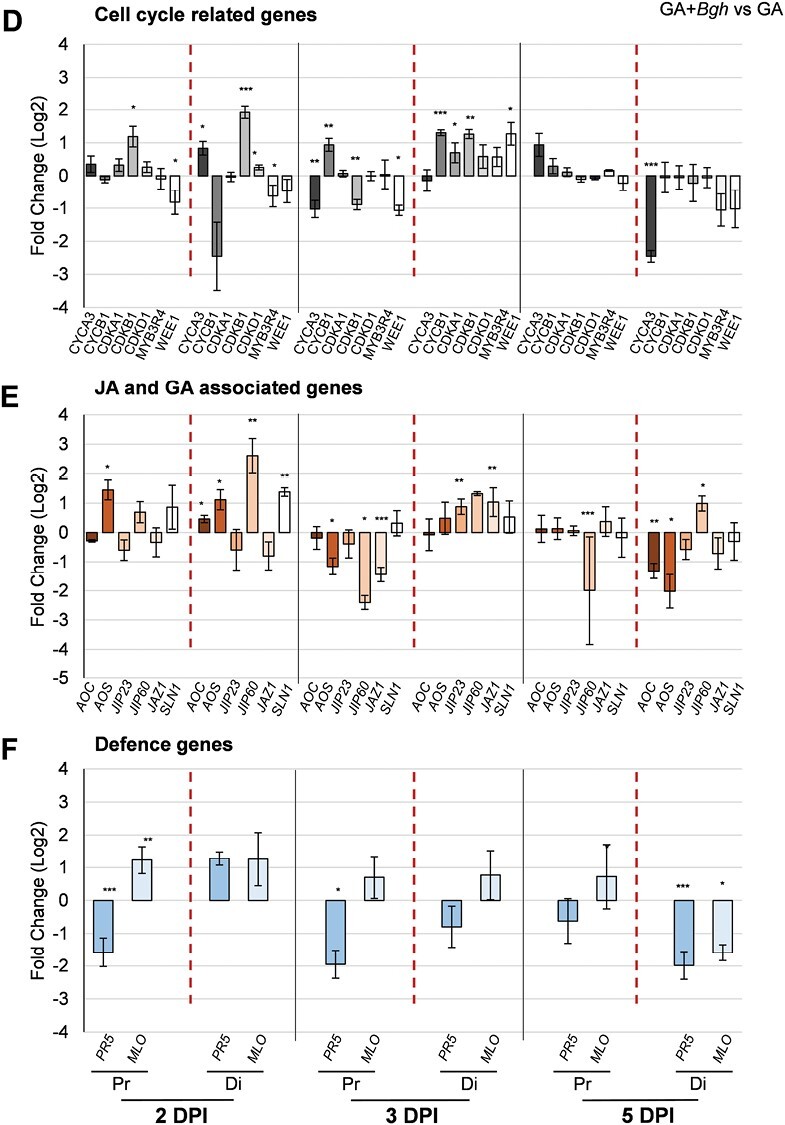
Antagonistic effects of MeJA and GA pre-treatment on the expression of genes associated with the cell cycle, JAs, GA, and plant defence in barley leaf blade following *Blumeria hordei* infection. Seedlings of barley Golden Promise were grown hydroponically. For the MeJA treatment, 50 µM MeJA was added to the solution at for 24 h at 5–6 days after sowing (DAS), and then the media substituted with 1/2 strength Hoagland’s solution No 2 and sampled at 7 DAS. For the GA treatment, 10 µM GA_3_ was present in the solution from the time of germination. At 7 DAS, leaves were detached and infected with *B. hordei*, and samples from the proximal (Pr) and distal (Di) sections taken 2, 3, and 5 days post-inoculation (DPI). Expression was determined using qRT-PCR with *GAPDH* as the reference gene. For each gene analysed, the results are presented as the fold-change in log_2_ expression in the hormone + *B. hordei* (*Bgh*) treatment compared with the corresponding sample from the hormone-only treatment. (A) Genes associated with the cell cycle, (B) genes associated with JA and GA, and (C) genes associated with plant defence. (A–C) Seedlings pre-treated with MeJA. (A) Genes associated with the cell cycle, (B) genes associated with JAs and GA, and (C) genes associated with plant defence. (D–F) Seedlings pre-treated with GA_3_. (D) Genes associated with the cell cycle, (E) genes associated with JAs and GA, and (F) genes associated with plant defence. Data are means (±SE) of three biological replicates, each of which consisted of samples pooled from three leaves. Significant differences in the fold-change in expression were determined using one-way ANOVA: *P<0.05, **P<0.01, ***P<0.001.

As MeJA pre-treatment reduced *B. hordei* infection in the proximal leaf blade (Fig. 4), the expression patterns of the same cell cycle, JA, and defence genes in infected leaf blades that were pre-treated with 50 µM MeJA for 24 h, was next examined, to gain insights into the phytohormone-mediated resistance mechanisms. Thus, the relative changes in gene expression were compared between MeJA pre-treated and *B. hordei-*infected proximal and distal leaf blades compared to the phytohormone treatment alone 2, 3, and 5 DPI (Fig. 7A–C).

Overall, the MeJA pre-treatment resulted in an increase in expression of cell cycle-related genes in *B. hordei*-infected leaf blades during the time-course, and this was particularly marked in the distal leaf blade (Fig. 7A). In particular, all the genes examined showed increased expression in the distal leaf blade at 5 DPI following the MeJA pre-treatment, with *CYCA3* increased by 2.8-fold (*P*=3.55 × 10^–6^), *CDKA1* (*P*=1.1 × 10^–5^), *CDKB1* (*P*=8.5 × 10^–3^), and *CDKD1* (*P*=2.0 × 10^–4^) increased by ~1.8-fold, and *CYCB1* increased by ~1.5-fold (*P*=2.6 × 10^–7^). *MYB3R4* and *WEE1* (*P*=9.8 × 10^–3^) showed the same effect as the canonical cell-cycle marker genes.

The expression of the JA-biosynthesis gene *AOS* (*P*=1.6 × 10^–2^) and the JA-inducible gene *JIP60* at 2 DPI was lower in the proximal portion of the leaf blade after MeJA pre-treatment and *B. hordei* infection than in the MeJA-only control (Fig. 7B). In the distal leaf blade, the expression of the two JA-biosynthesis genes and the two JA-inducible genes was higher than in the control at all the time-points (*AOC*, *P*=5.5 × 10^–3^; *JIP23*, *P*=2.2 × 10^–9^). Relative to the control, *AOS* and *AOC* expression was similar in the MeJA+*B. hordei*-treated distal leaf blade at 2 DPI and 3 DPI, but *AOC* expression was markedly higher at 5 DPI (*P*=4.6 × 10^–10^).

Relative to the control, the expression of *JIP23* (*P*=9.0 × 10^–4^), *JIP60*, and *JAZ1* (*P*=1.6 × 10^–2^) was higher in the distal leaf blade at 5 DPI in the MeJA+*B. hordei* treatment (Fig. 7B). In the case of the proximal leaf blade, the expression of *AOC* and *AOS* was unchanged at 5 DPI, compared with MeJA pre-treatment alone. The expression of *JIP23* and *JIP60* increased with time in MeJA+*B. hordei*-treated proximal leaf blades, and the same pattern was observed for *SLN1* in the distal leaf blade. The increase in the expression of JA-associated genes in response to sustained *B. hordei* infection following MeJA pre-treatment is indicative of the contribution of the latter to the attenuation of pathogen infection.

The expression of *PR5* and *MLO* in the distal leaf blade increased with from 2 DPI to 5 DPI in the MeJA+*B. hordei* treatment, whereas their expression remained unchanged in the proximal leaf blade (Fig. 7C).

Thus, combining MeJA pre-treatment with *B. hordei* infection resulted in expression patterns of cell cycle- and hormone-associated genes that provide evidence for different host responses compared with MeJA or *B. hordei* treatment alone.

The existence of crosstalk between JAs and GA (reviewed by Pérez-Salamó *et al*., 2019) prompted the analysis of gene expression in the leaf blade following GA pre-treatment and subsequent *B. hordei* infection (Fig. 7D–F). Relative to pre-treatment with GA alone, the expression of *CYCB1*, *CDKA1*, *CDKD1*, *MYB3R4*, and *WEE1* increased from 2 DPI to 3 DPI in the distal leaf blade of the GA+*B. hordei* treatment (Fig. 7D). In the proximal leaf blade, the expression of all the cell-cycle markers except for *CYCB1* and the expression of *MYB3R4* and *WEE1* was generally decreased or unchanged from 2 DPI to 3 DPI. The expression of *CYCA3* and *WEE1* in the proximal leaf blade increased from 3 DPI to 5 DPI. Overall, the expression of all the cell cycle-related genes decreased in the distal leaf blade from 3 DPI to 5 DPI in the GA+*B. hordei* treatment.

The expression of *AOS* (*P*=1.7 × 10^–2^), *AOC* (*P*=2.1 × 10^–4^), *JIP60* (*P*=4.9 × 10^–3^), and *SLN1* was high in the GA-pre-treated distal leaf blade during initial period of *B. hordei* infection at 2 DPI but it progressively declined with time during the infection process (Fig. 7E). Only *JIP60* expression was significantly higher in the GA+*B. hordei* treatment in the distal leaf blade at 5 DPI (*P*=2.9 × 10^–2^).

Similar to the genes related to the cell cycle and the hormones, the expression of the defence-related *PR5* and susceptibility factor *MLO* decreased with time from 2 DPI to 5 DPI in the distal leaf blade in the GA+*B. hordei* treatment (Fig. 7F). In the proximal leaf blade the expression of *PR5* was generally low at all the time-points, while the expression of *MLO* remained relatively unchanged. Contrary to our expectations, whilst *MLO* expression in the distal leaf blade increased with time in the MeJA+*B. hordei* treatment (Fig. 7C), it decreased with time in the GA+*B. hordei* treatment (Fig. 7F). Hence, MeJA and GA treatment might antagonistically regulate barley defence responses following *B. hordei* infection.

## Discussion

### Growth in barley leaves is spatially separated and differentially regulated by MeJA and GA

**Fig. 8.**
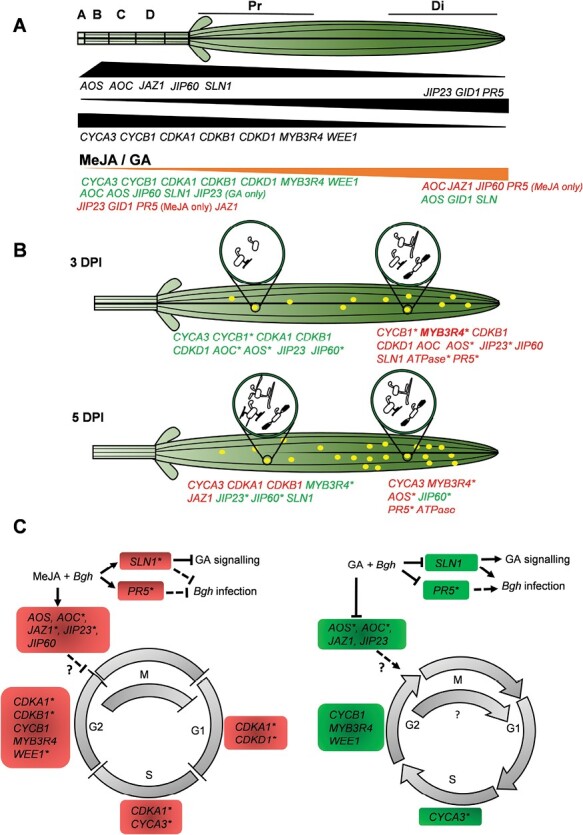
Diagrammatic summary to map the regulation of gene expression in barley leaf blade and sheath by phytohormones and *Blumeria hordei* infection. Gene induction is indicated in red and repression is indicated in green. Asterisks indicate changes in expression that were found to be significant in the current study. Dotted lines indicate an indirect or inferred process/mechanism. (A) In the absence of phytohormone treatment and infection, the expression of cell-cycle markers is generally higher in the sheath compared to the distal (Di) leaf blade (Fig. 3), and this is associated with higher nuclear DNA contents in the proliferating sheath tissue (Fig. 1), reflecting the different developmental stages in the two modules of the leaf. Expression of genes associated with JAs and GA is generally higher in the sheath and decreases towards the distal leaf blade, whereas *JIP23*, *GID1*, and *PR5* expression is higher in the distal section. The expression of *AOC*, *JAZ1*, and *JIP60* in the leaf blade increases following both MeJA and GA treatment. The expression of the cell cycle markers of *AOS* and *SLN1* is reduced by the two hormones in both the sheath and leaf blade. GA does not have any effect on the expression of *PR5*. (B) The effects of *B. hordei* on gene expression. The expression of *MYB3R4* (highlighted in bold) is already relatively high in the distal leaf blade prior to infection (Fig. 3), and together with *CYCB1* its expression increases significantly by 3 days post-inoculation (DPI; Fig. 5). The expression of the JAs-related genes *AOC*, *AOS*, *JIP23*, and *JIP60* as well as *SLN1* is also higher in the distal leaf blade at 3 DPI. The expression of *ATPase* and *PR5* is higher in the distal leaf blade at both 3 DPI and 5 DPI. *MLO* expression is higher only in the distal leaf blade at 3 DPI. At 5 DPI, *MYB3R4* and *AOS* expression increases in the distal leaf blade. In the proximal leaf blade, a noticeable, albeit not significant, increase in *CYCA3*, *CDKA1*, *CDKB1*, and *JAZ1* expression occurs at 5 DPI. Infection by *B. hordei* causes a reduction in the expression of *CYCB1* at 3 DPI in the proximal section and of *JIP60* in the distal section at 5 DPI. (C) Cumulative effects of pre-treatment with MeJA or GA combined with sustained *B. hordei* infection on cell cycle progression in the leaf blade (Fig. 7). Pre-treatment with MeJA negatively affects growth and arrests the cell cycle, affecting the transition checkpoints at G1/S and G2/M (Pauwels *et al.*, 2008; Noir *et al* 2013; Bömer *et al* 2018). The increase in expression of the JAs-associated genes *AOS*, *AOC*, *JAZ1*, *JIP23*, and *JIP60* might be associated with stabilization of the GA-signalling inhibitor *SLN1* following MeJA treatment and *B. hordei* infection. The increase in expression of *CYCB1* is in line with a previous study showing G2 arrest of Arabidopsis root cells and increased *CYCB1;1* expression after γ-irradiation (Ricaud *et al.*, 2007). The increased expression of *WEE1*, involved in the DNA damage response and G2 arrest, further reinforces the role of MeJA+*B. hordei* on cell cycle arrest. Pre-treatment with GA has the opposite effect to that of MeJA on cell cycle transitions and *B. hordei* infection in the leaf blade. The expression of *AOS*, *AOC*, *JAZ1*, and *JIP23* is reduced following GA pre-treatment and *B. hordei* infection, whilst the expression of *CYCA3*, *CYCB1*, *MYB3R4*, and *WEE1* is reduced. The reduction of *PR5* and *SLN1* expression following GA pre-treatment and *B. hordei* infection might contribute to increasing the susceptibility to *B. hordei*.

To identify and determine the extent of proliferation and endoreduplication along the barley leaf, flow cytometry was used to analyse the nuclear DNA content along the sheath and blade. A higher ratio of nuclei in the S/G2-M phase was observed in the sheath (Fig. 1B, C) and this was associated with elevated expression of cell-cycle genes (Fig. 3). Higher expression of *MYB3R4* and *WEE1* in the sheath reflected the presence of more proliferating and endoreduplicating cells, respectively, in the sheath compared to the leaf blade (Figs 3). A similar expression pattern for the inhibitory *WEE1* was observed by Beemster *et al*. (2005). *WEE1* might have a role in fine-tuning the cell cycle, inhibiting the mitotic cell cycle, and inducing endoreduplication/differentiation in response to environmental stress. The higher expression of the other markers associated with the cell cycle and proliferation could be reconciled within the global context of leaf development. In actively dividing meristematic cells, resources are predominantly directed into transcriptional regulation, cell proliferation, and protein synthesis to support cytoplasmatic growth (Noir *et al*., 2013; Nelissen *et al*., 2018; Loudya *et al*., 2021). Evidence of post-transcriptional regulatory mechanisms that are responsible for the differential activity of cell-cycle components is extremely limited in monocots. The trends that we observed for genes with a role in G2/M (such as *CYCB1* and *MYB3R4*), with steeper down-regulation in comparison with those with a role in G1/S (or both G1/S and G2/M) such as *CYCA3* and *CDKA*, were comparable to those previously observed in Arabidopsis (Beemster *et al*., 2005). Our results indicate spatial separation of growth-promoting processes in barley and are in line with previous observations in maize and wheat, where the cells transition from expanding to becoming mature (Nelissen *et al*., 2018; Loudya *et al*., 2021). Our study provides candidate targets for further investigations that are paramount for research in cereals (Fig. 8).

Treatment with MeJA reduced leaf elongation and the progression of the cell cycle (Fig 1E, F), matching observations in other species (Noir *et al*., 2013; Świątek *et al*., 2002, 2004; Pauwels *et al*., 2008; Bömer *et al*., 2018). The frequency of nuclei in the G2/M phase decreased after treatment, indicating that the cell cycle was mainly arrested in G1 prior to the S transition. MeJA has previously been found to repress the onset of endoreduplication in Arabidopsis (Noir *et al*., 2013); however, in our study, effects of MeJA treatment on the frequencies of 8C and 16C nuclear DNA contents in barley were not evident, in line with the lower occurrence of endoreduplication in monocots relative to dicots (Barow and Meister, 2003). In contrast to MeJA, GA promoted leaf elongation and increased, albeit slightly, nuclear DNA contents of 4C and above (Fig 1E, F). This is consistent with previously observed increased mitotic activity and positive effects on endoreduplication in other plant species (Perrazza *et al*., 1998; Fabian *et al.*, 2000; Yuxi *et al*., 2021). The opposing effects on the nuclear DNA content of MeJA and GA might reflect a broader control mechanism that balances growth by cell division and expansion.

Both JAs and GA regulate developmental switches that induce the transition from cell division to cell expansion. In this study, both phytohormones down-regulated the expression of genes associated with the cell cycle in the leaf (Figs 3, 7). Their effect was particularly evident in the basal region of the sheath (sections A, B in Fig. 1A) where cell division is more active. This phenomenon can be explained by the known negative effect of MeJA on cell-cycle activity (Świątek *et al*., 2004; Pauwels *et al*., 2008; Noir *et al*., 2013; Bömer *et al*., 2018) and with the role of GA in promoting cell differentiation and development at the expense of cell proliferation (Achard *et al*., 2009). In our study, the shift from cell division to differentiation was identified within the proximal sheath as supported by the strongest down-regulation of genes associated with the cell cycle by MeJA and GA. GA and JA interact synergistically and antagonistically with each other in seedling growth and resistance to pathogens through interactions between JAZ and DELLA proteins (Song *et al*., 2014). Such interactions suggest that JA-triggered growth inhibition could be fine-tuned by binding competition among JAZ, DELLA, and MYC2 proteins (Noir *et al*., 2013; Pérez-Salamó *et al*., 2019).

Higher expression of the JA-biosynthesis genes *AOS* and *AOC* in the sheath (Fig. 3) is in agreement with previous results for barley, where similar constitutive spatial and temporal expression has been observed (Maucher *et al.*, 2000, 2004). Higher expression levels of these JA biosynthesis genes also matches previously observed elevated content of JAs in lower portions of leaf blade sections (Maucher *et al.*, 2000, 2004). Similar hormone distribution along the growing maize leaf has been observed (Nelissen, 2018). The role of *JIP60* in seedling development is not known, but its coordinated expression alongside *AOS* and *AOC* might suggest it has a role in leaf growth regulation.

The lack of further *AOC* and *AOS* induction in the sheath by MeJA and GA might reflect the existence of a feedback mechanism that keeps tight control over cell proliferation and preservation of meristem identity in the sheath. In the leaf blade, *AOC* and *JIP60* expression are delayed compared to *AOS* (Maucher *et al.*, 2000, 2004). Whether this reflects the lack of activation of feedback mechanisms remains to be determined. The induction of *PR5* in response to MeJA, correlating with that of *AOC*, *JAZ1* and *JIP60* suggests a differential ability to activate defence responses in the leaf blade relative to the sheath.

Interestingly, higher GA levels have also been reported in proximal leaf sections alongside JAs (Nelissen, 2018). The patterns of expression of *SLN1* and *GID1* (Fig. 3), however, were not in apparent agreement with our expectations. Active GA signalling via the GID1 receptor would result in degradation of DELLA proteins/SLN1, leading to the activation of the GA response (Qi *et al*., 2014). The decrease in expression of *SLN1* in response to MeJA and GA might correlate with the induced expression of *JAZ1* and/or the JA induction of *GID1*. However, as reports on the transcriptional regulation of *SLN1* and *GID1* are sparse, it remains to be determined whether the expression patterns observed in our work, correlate with post-translational regulation of GA signalling components. Overall, the MeJA and GA expression signatures in the leaf reflected hormone crosstalk to maintain the cell metabolic homeostasis.

### Distinct gene expression patterns are associated with both *B. hordei* infection strategy and host responses

*Blumeria hordei* is an obligate biotrophic pathogen that proliferates rapidly on the leaf surface (Both *et al.*, 2005), thereby imposing metabolic demands on the host (Chandran *et al*., 2010, 2013). The molecular and genetic interactions of this pathogen with both host (Kuhn *et al.*, 2016) and non-host plants (Lipka *et al*., 2005; Kusch and Panstruga, 2017; Spanu and Panstruga, 2017; Panstruga and Moscou, 2020) have been extensively studied. In Arabidopsis, infection by another powdery mildew, *Golovinomyces orontii*, is correlated with the occurrence of endoreduplication, which potentially promotes the metabolic capacity of host cells at the infection site (Chandran *et al*., 2010). We therefore examined whether *B. hordei* induced endoreduplication in the barley leaf blade, and our results showed that epidermal cells of infected leaves had increased epidermal nuclear size but did not have any alteration in their nuclear DNA ploidy distribution (Fig. 6; Supplementary Fig. S2), suggesting that infection does not necessarily induce endoreduplication in the barley leaf blade. It is possible that *B. hordei* might alter the levels of nuclear envelop proteins and hence remodel the nuclear morphology, thereby affecting nuclear transport and signalling, and ultimately immune responses (Goto *et al*., 2021).

Remarkably, the cell-cycle genes examined, showed differential expression patterns in the proximal and distal leaf blade, and this was associated with different levels of hyphae formation in these sections (Figs 4B, D, 5A, 8B). The repression of cell-cycle genes in the proximal leaf blade early during infection suggests that the pathogen might suppress cell proliferation, thereby triggering cell differentiation. Whether this is associated with metabolic reprogramming that satisfies the requirement of the biotrophic *B. hordei* remains to be determined. Alternatively, it could indicate the occurrence of a host defence mechanism to halt growth in response to *B. hordei*. During infection, the up-regulation of genes for cell-cycle progression could reflect a release from *B. hordei*-induced stress. This would be consistent with the absence of induction of the genotoxic stress-related *CYCB1* (Ricaud *et al*., 2007). On the other hand, the greater susceptibility of the distal leaf blade to sustained *B. hordei* infection (i.e. 5 DPI) was associated with lower expression of genes for cell-cycle progression and higher expression of *MYB3R4* (Fig. 5A), implying a further attempt of the pathogen to drive cell differentiation (Haga *et al*., 2007, 2011) to its advantage for successful infection. In addition, *CYCB1* down-regulation over time could be the result of reduced host perception of *B. hordei*-induced stress, perhaps as a consequence of pathogen activity.

JA signalling plays a vital role in plant defences (Pérez-Salamó *et al*., 2019). The increased expression of all the JA-associated genes examined, as well as that of *SLN1*, in the distal leaf blade following *B. hordei* infection 3 DPI (Figs 5B, 7B), indicated the activation of host stress responses concomitant with modulation of growth. These observations are in agreement with results previously obtained in both monocots and dicots, where pathogen infection has been shown to increase the expression of *AOS*, *AOC*, and downstream JA-signalling components such as *JAZ1*, *COI1*, *PR14*, *JIP60*, and *MYB* (Maucher *et al.*, 2000, 2004; Polesani *et al.*, 2010; Cao *et al.*, 2015; Guerreiro *et al.*, 2016). Conversely, the reduced expression of some JA biosynthesis and signalling genes at 5 DPI might have been the result of pathogen suppression of the host defence response following its establishment. Thus, *B. hordei* might have evolved strategies to repress JA signalling, possibly through induction of the antagonistic SA (Achuo *et al*, 2004), and perhaps to promote GA signalling in order to facilitate exploitation of the barley leaf cellular machinery.

H^+^-ATPases are plasma-membrane (PM) pumps that establish cellular membrane potential in plants, which is crucial for controlling many transport processes (Palmgren, 2001). These pumps are actively regulated during plant immune responses and are important targets of pathogens (reviewed in Elmore and Coaker, 2011). Infection of tomato with *Cladosporium fulvum* and of barley with *B. hordei* increases H^+^-ATPase protein levels, resulting in the generation of proton gradients between the apoplast and the extrahaustorial space (Vera-Estrella *et al.*, 1994; Szabo and Bushnell, 2001). Thus, manipulation of ATPases in the plasma membrane/apoplast by *B. hordei* might benefit its growth through increased nutrient transport. This is substantiated by the findings of Lambertucci *et al.* (2019), where increased abundance of V-type PM-ATPase protein levels following *B. hordei* infection was observed.

Increased expression levels of the well-characterized susceptibility gene *MLO* (Kusch and Panstruga, 2017; Devoto *et al*., 1999) in the distal leaf blade at 3 DPI coincided with the establishment of the infection; however, the establishment of the host response might be reflected by its lack of induction at 5 DPI (Figs 4B, D, 5C). Higher expression of *PR5* correlated with *B. hordei* infection in the distal leaf blade, which agrees with previous observations by Lambertucci *et al*. (2019). The extent of *B. hordei* infection along the leaf blade might therefore be affected by the gradient of expression of JA-related and defence genes, contributing to shielding the proliferating cells in the sheath from the invading pathogen. Taken together, our results suggest that blocking or interfering with these *B. hordei* infection strategies could be used as a strategy to limit powdery mildew disease in barley and other plants.

### Pre-exposure to MeJA or GA differentially regulates defence responses along the leaf blade

Stress-signalling hormones can orchestrate gene priming or transcriptional memory (Liu and Avramova, 2016). In Arabidopsis, MeJA treatment induces priming of the plant stress response (Noir *et al*., 2013), and it triggers developmental and stress responses generally in plants (Wu *et al.*, 2008; Pérez-Salamó *et al.*, 2019); however, the exact mechanism by which JAs modulates the responses to pathogen infection is not fully understood. Here, we attempted to examine the effects of exogenous MeJA as well as GA on *B. hordei* infection of barley leaf blade, in terms of the expression of genes related to the cell cycle and defence. The classical model for infection resistance states that the JA pathway is activated during necrotrophic infection (Díaz, *et al.*, 2002; Ferrari *et al.*, 2003; Zarate *et al.*, 2007); however we found that exogenous MeJA also seemed to play a role in response to the biotrophic infection by *B. hordei*. This is consistent with a report that MeJA treatment protects Arabidopsis against the biotrophic pathogens *Erysiphe cichoracearum* and *Peronospora parasitica* (Zimmerli *et al.* 2004).

The localized up-regulation of cell cycle progression genes in the distal leaf blade following MeJA pre-treatment and subsequent *B. hordei* infection (Fig. 7A) could have resulted from host attempts to trigger a defence response, in agreement with other studies showing stress-inducibility of genes associated with the cell cycle (Ricaud *et al*., 2007, Vanderauwera *et al*., 2011; Waterworth *et al*., 2016; Shimotohno *et al*., 2021). The enhanced susceptibility of the distal leaf blade to *B. hordei* could underlie the effect of the pathogen to counteract the host response, exemplified by the induction of the growth repressor *WEE1*. This was substantiated by the higher up-regulation of the stress marker *CYCB1* in the distal portion of the leaf blade compared to the proximal one. *CYCB1* plays a crucial role in genotoxic stress responses, production of reactive oxygen species, and double-strand breaks in host-plant DNA following bacterial, fungal, or oomycete infection (Veylder *et al.*, 2007; Song and Bent, 2014; Weimer *et al.*, 2016; Chen *et al.*, 2017). The effect of MeJA pre-treatment on the expression of this class of genes during infection was not evident in the less-infected proximal leaf blade, highlighting the occurrence of different defence mechanisms along the leaf blade. Whether this can be accounted for by differences in cell development status and competence to respond to MeJA treatment along the leaf blade remains to be determined.

We also examined the expression of JA-associated genes in leaf blades pre-treated with MeJA and then infected with *B. hordei*, and we found that MeJA pre-treatment significantly increased their expression during infection (Fig. 7B). The activation of the complex ribosome-inactivating protein *JIP60* in the MeJA pre-treated leaf blade agrees with its role in JA-dependent plant immunity (Przydacz *et al*., 2020). Overall, the expression of JAs related genes increased with time, albeit with different levels of magnitude in the proximal and distal leaf sections. The reduced *B. hordei* infection in the proximal section (Fig. 4D) was associated with a stronger induction response of these genes that might have resulted in more effective activation of stress signalling than in the distal section where the expression was higher to start with. The increased expression of the DELLA gene *SLN1* following infection suggested a possible concurrent reduction in GA signalling and an increase in JA signalling. Whether such a mechanism is implemented to prioritize defence over growth by suppressing the GA signalling cascade in barley remains to be determined.

The expression of *PR5* was significantly increased by MeJA pre-treatment plus *B. hordei* in the distal leaf blade at 5 DPI (Fig. 7C), similar to its induction following *B. hordei* infection alone (Fig. 5). However, the MeJA pre-treatment might have delayed the onset of the infection, as evidenced by the later induction of *PR5.* The absence of induction of *PR5* in the proximal section might reflect the existence of lower susceptibility to infection in this section of the leaf (Fig. 4).Overall, our results suggested that MeJA-induced priming might contribute to the activation of defence responses in barley leaf cells, although it was notable that the mechanism was differently executed along the length of the leaf blade.

JAs play the main role in plant defence responses, while GAs predominantly regulate plant growth and development. Hence, JA-GA crosstalk finely balances the trade-off between growth and stress responses (Hou *et al.*, 2010; Pérez-Salamó *et al*., 2019). The antagonistic effects of JAs and GAs on each other’s signalling output to balance growth and defence has been reported previously (Yang *et al.*, 2012, 2019; Um *et al.*, 2018). The down-regulation of the JA-related genes, the DELLA *SLN*, the stress-marker *CYCB1*, and defence-related *PR5* by pre-treatment with GA followed by *B. hordei* infection in the distal leaf blade (Fig. 7D–F) was indicative of a GA-mediated reduced ability to perceive the stress whilst continuing growth, leading to enhanced susceptibility relative to the proximal section (Fig. 4E). The opposite expression patterns that were observed for the different categories of genes in the infected plants following pre-treatment with JA or GA (Fig. 7) implies a tight control on growth regulation by the two phytohormones during the pathogen infection.

## Conclusions

Our study has demonstrated that the barley sheath and the leaf blade possess different growth capacities, intrinsic abilities to respond to hormones, and susceptibilities to *B. hordei* infection. The differential growth capacity is further fine-tuned by MeJA and GA by regulating genes related to the cell cycle, hormones, and defence. Different gene expression patterns along the barley leaf blade are also associated with both the *B. hordei* invasion strategy and host responses during sustained infection, and are further modified by priming with the two hormones in an antagonistic manner (Fig. 8). Ultimately, our results suggest that tight control is operated on cell proliferation and the preservation of meristem identity to guarantee plant survival.

Overall, the individual and the combination of treatments applied, as well as the analysis of leaf sheath and blade sections, has demonstrated the need for a more systematic approach to understand the underlying mechanisms that regulate the growth–stress trade-off. Identification of the regulators of the differential spatial responses to pathogens in the leaf and the correlation with growth processes will be instrumental for engineering plant resistance to *B. hordei* and other pathogens that show organ, tissue, and cell specificity.

## Supplementary data

The following supplementary data are available at *JXB* online.

Fig. S1. Ploidy levels in the sheath compared to the leaf blade in barley cultivar Haruna Nijo.

Fig. S2. Ploidy distribution frequencies in proximal and distal sections of leaves during infection by *B. hordei*.

Table S1. Primers used for qRT-PCR analysis.

Table S2. Frequencies of nuclei exhibiting 2C–16C DNA content in sheath and leaf of the barley cultivar Haruna Nijo.

Table S3. Frequencies of nuclei exhibiting 2C–16C DNA content in the proximal and distal sections of leaves of the barley cultivar Golden Promise.

erad331_suppl_Supplementary_Figures_S1-S2_Tables_S1-S3

## Data Availability

All data supporting the findings of this study are available within the paper and within its supplementary data published online.
