# Supplementary material for: Jasmonates, gibberellins, and powdery mildew modify cell cycle progression and evoke differential spatiotemporal responses along the barley leaf
Source: J Exp Bot. 2023 Aug 23;75(1):180–203. doi: 10.1093/jxb/erad331 (PMC10735486; doi:10.1093/jxb/erad331)
Supplement: erad331_suppl_Supplementary_Figures_S1-S2_Tables_S1-S3 [file erad331_suppl_supplementary_figures_s1-s2_tables_s1-s3.pdf]

Full Title

Jasmonates, gibberellins and powdery mildew modify cell cycle progression and evoke differential spatiotemporal responses along the barley leaf

Jovaras Krasauskas[1,4#], Showkat Ganie[1#], Aroub Al-Husari[1], Laurence Bindschedler[1], Pietro Spanu[2], Masaki Ito[3] and Alessandra Devoto[1]

[1] *Plant Molecular Science and Centre of Systems and Synthetic Biology, Department of Biological Sciences, Royal Holloway University of London, Egham, Surrey, TW20 0EX, United Kingdom*

[2] *Department of Life Sciences, Imperial College London, London SW7 2AZ, United Kingdom*

[3] *School of Biological Science and Technology, Kanazawa University, Ishikawa 920-1192, JAPAN*

[4] Present Address: The John Innes Centre, Norwich Research Park, Norwich, Norfolk, NR4 7UH, *United Kingdom*

#These authors contributed equally to the article

*** To whom correspondence may be addressed**

Alessandra Devoto (Alessandra.Devoto@rhul.ac.uk)

Information on Supplemental information

Supplementary Figures S1-S2

Fig. S1. Ploidy levels in the sheath compared to the leaf blade in barley cultivar Haruna Nijo

Fig. S2. Ploidy distribution frequencies in proximal and distal sections of leaves during infection by B. hordei.

Table S1. Primers used for qRT-PCR analysis

Supplemental Table 1. Frequency of nuclei exhibiting 2C-16C DNA content in barley Haruna Nijo sheath and leaf

Supplemental Table 2. Primers used for QRTPCR analysis.

Supplemental Table 3. Frequency of nuclei exhibiting 2C-16C DNA content in barley Golden Promise proximal and distal leaf

Supplemental Figures

Figure S1

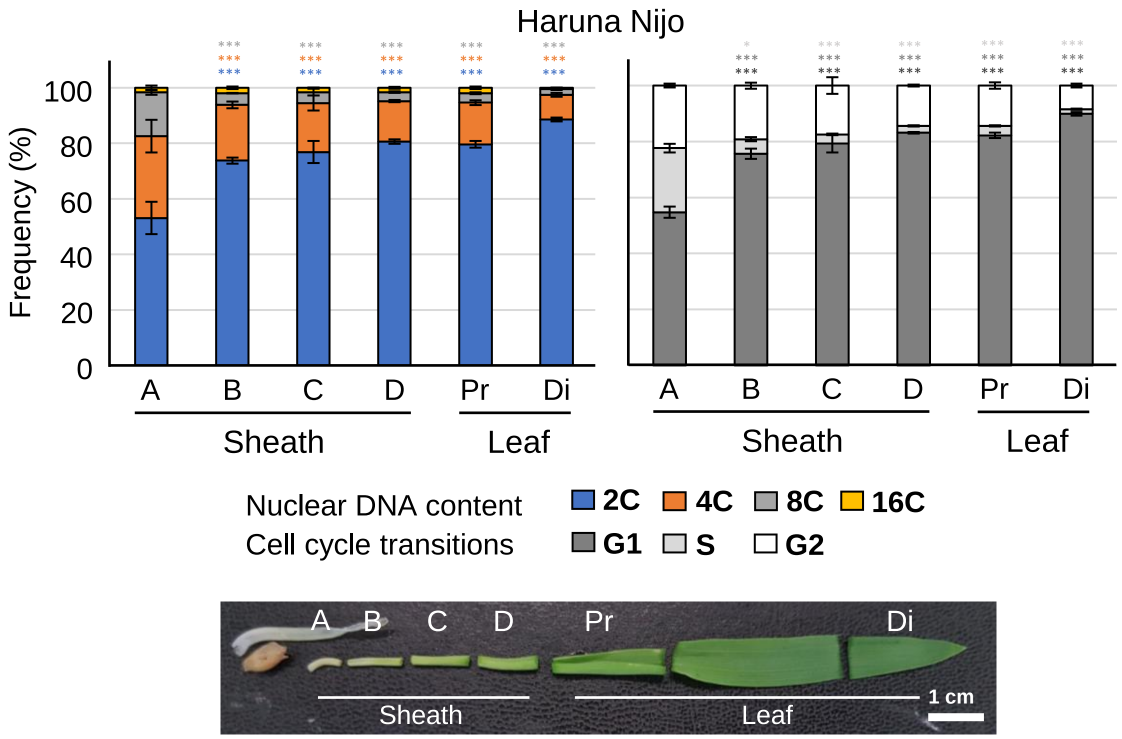

Ploidy levels are higher in the sheath compared to the leaf in Haruna Nijo barley cultivar. 7 DAS Haruna Nijo seedlings were collected, and the primary leaf dissected into smaller segments of 5 to 10 mm from the seed; A: 0-5 mm, B: 5-15 mm, C: 15 -20 mm and D: 20-25 mm, and 2 cm in the Proximal (Pr) and Distal (Di) leaf blade. The values represent average frequencies of the observed ploidy (or C) levels. Higher nuclear DNA ploidy was observed in the sheath of HN in comparison to the leaf blade (top panel). Cell cycle transitions mimicked the nuclear DNA ploidy trend; where higher S/G2-M phase was observed in the sheath, specifically in closer proximity to the meristem (segment A of the sheath) in HN. n = 15,000-20,000 nuclei counted from 3 biological replicates, 3-4 seedlings per experiment, with 2 technical replicates. Error bars ±SD). One-way ANOVA, with Tukeys post-hoc, analysis was used to analyse the differences in nuclear DNA content between Haruna Nijo sheath and leaf. Statistical analysis was performed using GraphPad Prisma and Xrealstats add-in in Microsoft Excel.

Figure S2

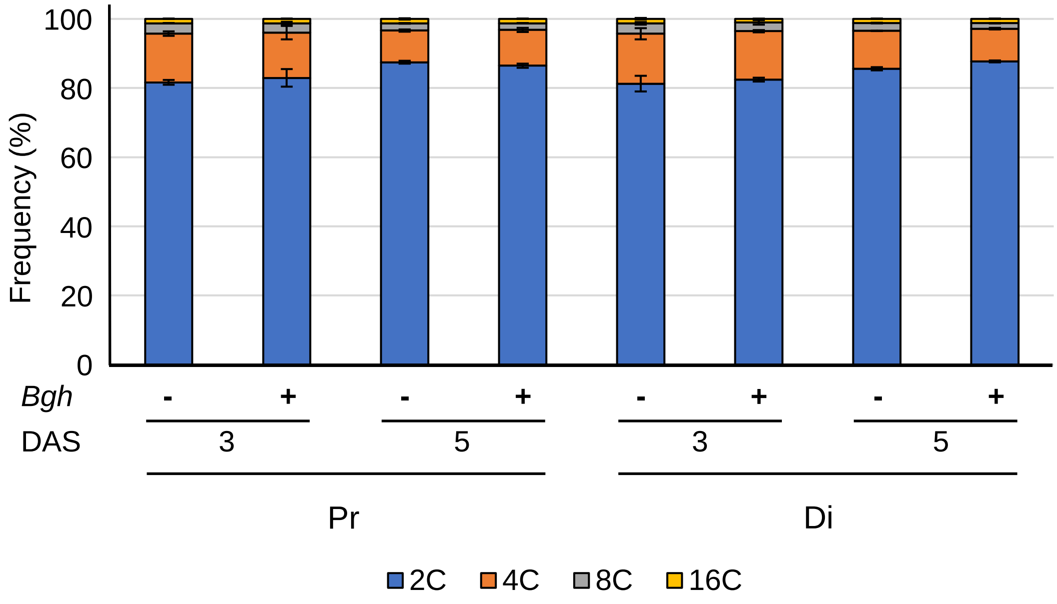

Bgh infection does not alter the overall ploidy distribution in the infected barley leaf. Flow cytometry analysis performed on Bgh infected barley leaf after 3- and 5-days post infection. No changes in nuclear DNA ploidy in the infected leaf in comparison to the control were observed (P=proximal, Di=distal). n = 15,000-20,000 nuclei counted from 3 biological replicates, 3-4 seedlings per experiment, with 2 technical replicates. Error bars ±SD. One-way ANOVA, with Tukeys post-hoc, analysis was used to analyse the differences in nuclear DNA content between *Bgh* infected leaf and uninfected at 3 and 5 DPI. Statistical analysis was performed using GraphPad Prisma and Xrealstats add-in in Microsoft Excel.

Supplemental Tables

Supplemental Table 1. Primers used for qRTPCR analysis.

Gene	Gene ID	Forward (5’-3’)	Reverse (5’-3’)
AOC	HORVU.MOREX.r2.6HG0514200.1	TCGTCCCCTTCACCAACAAG	CTGTAGATGGCCTCGTAGCG
AOS	HORVU.MOREX.r2.4HG0328610.1	TCCTCGCTCCAATCTTGTCT	GCCGCTCTGGTTCATCTAAC
ATPase	HORVU.MOREX.r2.2HG0168500.1	ACATCGACACCATCAACCAA	ACAACTAGGGGCTGGTCAGA
CDKA1	HORVU.MOREX.r2.6HG0457700.1	GGTGGTAACATTATGGGACAGAG	TTCAGCAAAAATGCAACCAA
CDKB1	HORVU.MOREX.r2.7HG0565000.1	ACACGCACGAGATCCTTACT	CAAATATGCAGCCCACAGACC
CDKD1	HORVU.MOREX.r2.1HG0051370.1	TGGTCTTGCACGCATATTCG	ATACAACCAGCAGCCCAAAC
CYCA3	HORVU.MOREX.r2.5HG0363660.1	GAAGACAAGAAGCGCTCCAG	GATGAAGCTGCAACCACTGA
CYCB1	HORVU.MOREX.r2.3HG0246040.1	GCTCCTGAGGTGAATGATTTC	GGTGTTCTCCATCTCCTTGTC
GAPDH	2H:630028086:630034599:1	CTGATTGAGAAGGCTGATGGAT	AGAGCAGGAGCGTCATTGA
GID1	HORVU.MOREX.r2.1HG0049800.1	AGAGCCTCATCATCGTGTCC	TTGGAGAGCAGGTAGAAGCC
JAZ1	HORVU.MOREX.r2.2HG0103320.1	GAATCAACAAGGGTGAGGCC	ATGGTCTCCTTCTTCCTGCC
JIP23	HORVU.MOREX.r2.6HG0512860.1	TGTTGCAGACTATGCCATGAA	TGCCAATCGTTGTACTTAGCC
JIP60	HORVU.MOREX.r2.4HG0345940.1	GCTCAACTACCCAAACACGG	CCGGGCTGTAAATGAAGTCG
Mlo	HORVU.MOREX.r2.4HG0342080.1	ACCGTCCAGGAAGATGTCTG	TCTGCATACAAAGCCAGCAC
MYB3R4	HORVU.MOREX.r2.3HG0206530.1	CGACGCTCAACCAAAGGAAA	GCAGGCATTGTACATCGGTT
PR5	HORVU.MOREX.r2.5HG0351950.1	CACGGACATCACCAAGGATT	TTGCCCTTGAAGAACATTGAG
SLN1	HORVU.MOREX.r2.4HG0280720.1	TCAGTCTTCGAGATGCACCG	TGAAGCGGTCCAGGAATGAG
Ubiquitin	1H:500272516:500273449:1	CAGTAGTGGCGGTCGAAGTG	ACCCTCGCCGACTACAACAT
WEE1	HORVU.MOREX.r2.6HG0461230.1	CTGCCCCGAGAGAAAAGAT	GCTTGGAGATTTCGGTTGTG

Cell cycle markers *CYCB1*, *CYCA3*, *CDKA1*, *CDKB1*, *CDKD1* and *WEE1* were identified in(Gendreau et al., 2008; Gendreau et al., 2012).

The forward primers were taken from the publication, and the reverse primers were designed using Primer3 to obtain ~120-200 bp amplicon lengths for qRT-PCR.

JAZ, *AOS*, and *AOC* were identified in barley through homologous search in Arabidopsis and rice genomes.

GAPDH and *MLO* primer sequences were provided by Dr. L. Bindschedler RHUL.

ATPase, *Ubiquitin*, *PR5*, *JIP23* and *JIP60* primers were identified in the literature (Visnovitz et al., 2012; Schäfer et al., 2009; Rustgi et al., 2014). *GID1* and *SLN1* identified in (Chandler et al., 2002; Dockter & Hansson, 2015), and primers designed using Primer3. All primers have the annealing temperature of 60 ^{o}C.

Supplemental Table 2. Frequencies of nuclei exhibiting 2C–16C DNA content in sheath and leaf of the barley cultivar Haruna Nijo.

	A	B	C	D	Pr	Di
2C	53.10±5.86	73.41±1.07	76.85±3.99	80.93±0.79	79.28±0.13	88.75±0.37
4C	29.49±5.87	19.97±1.20	17.65±2.72	14.65±0.43	14.95±0.86	8.87±0.70
8C	15.73±0.82	4.18±0.13	3.89±1.21	3.20±0.29	3.35±0.33	2.06±0.31
16C	1.66±0.83	1.92±0.40	1.58±0.12	1.60±0.32	1.98±0.46	0.46±0.06
G1	54.72±2.03	75.65±1.84	79.30±3.23	83.17±0.29	82.18±0.94	89.89±0.60
S	22.94±1.53	5.15±0.77	3.21±0.27	2.40±0.27	3.37±0.25	1.60±0.21
G2	22.33±0.65	19.19±1.09	17.48±2.95	3.00±0.36	14.44±1.13	8.49±0.66

The values represent nuclear DNA content (C) level fractions in Haruna Nijo, as illustrated in Supplementary Figure 1.

The analyses were performed on at least 15,000 nuclei isolated from three sheath/leaves for each ploidy measurement.

The sheath was dissected into 5-10 mm segments from the meristem (seed), and 20 mm in proximal and distal leaf.

Flow cytometry experiments were repeated at least three times for each independent biological replicate.

Supplemental Table 3. Frequencies of nuclei exhibiting 2C–16C DNA content in the proximal and distal sections of leaves of the barley cultivar Golden Promise.

DAS	3				5			
Bgh	-	+	-	+	-	+	-	+
	Proximal		Distal		Proximal		Distal	
2C	86.33±0.71	82.29±2.53	86.86±0.59	86.81±0.62	85.62±2.23	82.70±2.23	80.23±0.48	87.51±0.48
4C	9.07±0.78	12.98±1.95	10.28±0.44	10.38±0.64	11.01±1.61	14.05±1.61	14.26±0.03	9.42±0.03
8C	2.05±1.24	2.70±0.46	1.92±0.17	1.93±0.09	2.27±0.35	2.54±0.35	2.94±0.65	1.64±0.10
16C	1.30±0.59	1.26±0.05	1.45±0.16	1.27±0.05	1.19±0.24	1.01±0.24	1.31±0.05	1.21±0.05

The values represent nuclear DNA content (C) level fractions as illustrated in Supplementary Figure 2.

The analyses were performed on at least 15,000 nuclei isolated from three sheath/leaves for each ploidy measurement.

7 DAS seedlings were infected with *Bgh* and samples collected for analysis at 3 DPI and 5 DPI.

Flow cytometry experiments were repeated at least two times for each independent biological replicate.
